# GPR162 activates STING dependent DNA damage pathway as a novel tumor suppressor and radiation sensitizer

**DOI:** 10.1038/s41392-022-01224-3

**Published:** 2023-02-01

**Authors:** Yao Long, Jiaxing Guo, Jielin Chen, Jingyue Sun, Haiyan Wang, Xin Peng, Zuli Wang, WeiWei Lai, Na Liu, Long Shu, Ling Chen, Ying Shi, Desheng Xiao, Shuang Liu, Yongguang Tao

**Affiliations:** 1 Department of Pathology, Key Laboratory of Carcinogenesis and Cancer Invasion(Ministry of Education), Xiangya Hospital, Central South University, Changsha, Hunan 410078 China; 2 NHC Key Laboratory of Carcinogenesis of Ministry of Health (Central South University), Cancer Research Institute, School of Basic Medicine, Central South University, Changsha, Hunan 410078 China; 3 Department of Pathology, Xiangya Hospital, Central South University, Changsha, Hunan 410008 China; 4 Hunan Key Laboratory of Tumor Models and Individualized Medicine, Department of Thoracic Surgery, Second Xiangya Hospital, Central South University, Changsha, China; 5 Hunan International Scientific and Technological Cooperation Base of Brain Tumor Research, Xiangya Hospital, Central South University, Changsha, Hunan 410008 China

**Keywords:** Cancer genetics, Cell biology

## Abstract

In the treatment of most malignancies, radiotherapy plays a significant role. However, the resistance of cancer cells to ionizing radiation (IR) is the main reason for the failure of radiotherapy, which causes tumor recurrence and metastasis. In this study, we confirmed that GPR162, an orphan receptor in the G-protein-coupled receptor family, acted as a novel radiotherapy sensitizer by interacting with the stimulator of interferon genes (STING), which targeted DNA damage responses, activated IRF3, accelerated the activation of type I interferon system, promoted the expression of chemokines including CXCL10 and CXCL4, and inhibited the occurrence and development of tumors. Interestingly, the activation of STING by overexpression of GPR162 was independent of the classical pathway of cGAS. STING inhibitors could resist the antitumor effect of overexpression of GPR162 in IR-induced mouse models. In addition, most solid tumors showed low expression of GPR162. And the higher expression of GPR162 indicated a better prognosis in patients with lung adenocarcinoma, liver cancer, breast cancer, etc. In summary, these results suggested that GPR162 may serve as a potential sensitizer of radiotherapy by promoting radiotherapy-induced STING-IFN production and increasing the expression of chemokines including CXCL10 and CXCL4 in DNA damage response, providing an alternative strategy for improving cancer radiotherapy.

## Introduction

Radiotherapy is a treatment for many malignancies and provides effective relief of patients’ tumor-related symptoms.^1^ Ionizing radiation (IR) and chemicals can cause DNA double-strand breaks, which can be especially deadly to tumor cells.^2^ However, recent studies have revealed that traditional cancer therapies such as radiotherapy and chemotherapy can also stimulate anti-tumor immunity, which is critical to the treatment’s efficiency.^3,4^ For example, radiation-induced tumor cell micronucleus activates the cytoplasmic nucleic acid sensor pathway cGAS-STING.^5,6^ Stimulator of interferon genes (STING) is an integral endoplasmic reticulum (ER)-membrane protein. When STING senses DNA that shouldn’t be present in the cytoplasm, it can activate STING and further activate TBK1, induce phosphorylation of the transcription factor IRF3 into the nucleus, and produce type I interferons (IFN) and cytokines, and then activate innate immunity.^7–9^

In previous studies, we found that Lymphoid-specific helicase (LSH) plays a crucial part in the progression of cancer, which has major implications for the development of novel strategies to treat cancer.^10–16^ We have confirmed that LSH can mediate p53 to regulate ferroptosis and apoptosis of tumor cells.^17,18^ In our research, we found that LSH is a key molecule that regulates p53-related lncRNA (P53RRA).^19,20^ In addition, GPR162 is abnormally lowly expressed in lung cancer cell lines overexpressing P53RRA, but its relationship with LSH is not yet known. As is known to all, cancer-promoting gain-of-function activities can be induced by mutant p53 (mtp53) proteins.^21^ Research reports, that the cytoplasmic DNA sensing machinery, cGAS-STING-TBK1-IRF3, which stimulates the innate immune response, is disrupted by mtp53.^21^ But whether there is a link to GPR162 has not been reported.

GPR162 is a class A, rhodopsin-like G protein-coupled receptor (GPCR).^22^ Studies have reported that GPR162 is broadly expressed in GABAergic and other neurons, especially in regions associated with energy balance and hedonic feedings, such as the hypothalamus, amygdala, and ventral tegmental areas. Furthermore, variants of the GPR162 gene were linked to glucose homeostasis abnormalities, according to human genetic research.^23^ But the role of GPR162 in tumors and its mechanism need to be further explored.

In this study, we proved that GPR162 could promote the DNA damage response induced by radiotherapy, and release DNA from the nucleus into the cytoplasm to activate STING and further activate the transcription of type I IFN genes. We proved that the activation of the STING-related signal pathway by GPR162 is independent of the classical pathway of cGAS but directly acts on STING. More importantly, GPR162 can enhance the therapeutic effect of RT through STING to further inhibit the occurrence and development of tumors. These findings provide a new direction for the development of targeted treatment strategies that utilize the role of GPR162 in radiotherapy.

## Results

### GPR162 interacts with STING in mitochondria and endoplasmic reticulum

**Fig. 1:**
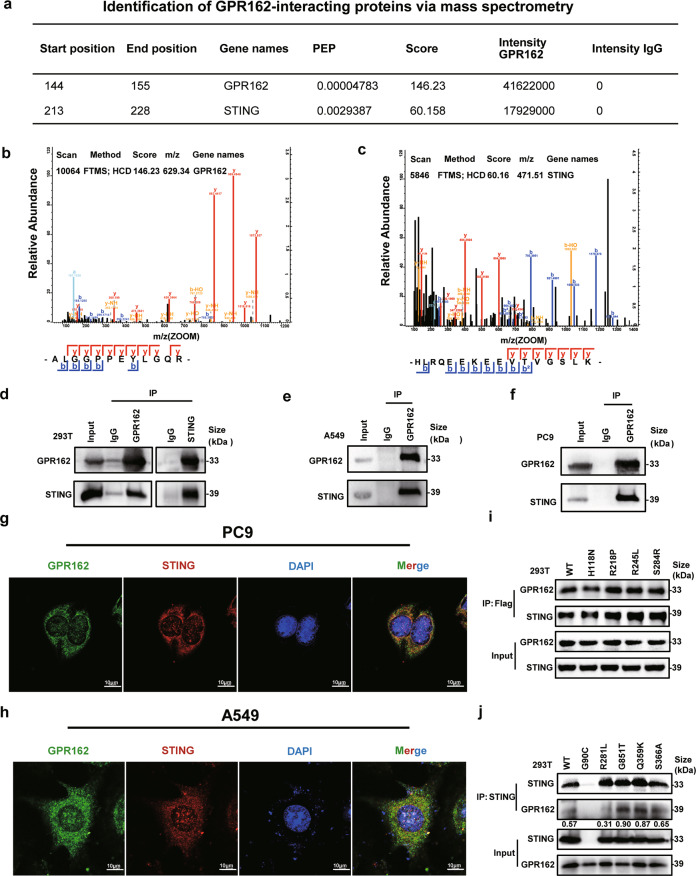
GPR162 interacts with STING in mitochondria and endoplasmic reticulum. **a**–**c** Mass spectrometry analysis shows a protein interaction between GPR162 and STING. **d** GPR162 and STING were simultaneously transfected into 293T cells, and the interaction between GPR162 and STING was detected by Co-IP analysis, IgG was the negative control. **e**, **f** IP identification of endogenous GPR162 and STING interactions in A549 (**e**) and PC9 (**f**) parent cells, IgG was the negative control. **g**, **h** Confocal microscopy images of A549 (**h**) and PC9 (**g**) stained with anti-GPR162, anti-STING antibodies, and DAPI. Scale bar, 25 μm. **i** The interaction between GPR162 and STING was detected by IP analyses in 293T cells transfected with GPR162 WT and mutant plasmids including H118N, R218P, R245L, and S284R. **j** The interaction between GPR162 and STING was detected by IP assays in 293T cells transfected with STING WT and STING mutants including G90C, R281L, G851T, Q359K, and S366A

We found that GPR162 is negatively correlated with LSH mRNA and protein expression levels (Supplementary Fig. 1), suggesting that it is very significant in tumors. Protein mass spectrometry was used to further undermine the mechanism of GPR162 in tumors (Supplementary Fig. 2a). Mass spectrometry analysis showed that STING was immunoprecipitated by GPR162 (Fig. 1a–c, Supplementary Table 1), which aroused our great interest, and the interaction between GPR162 and STING was confirmed by immunoprecipitation analysis endogenous and exogenous (Fig. 1d–f). Next, we predicted through the website (https://www.genecards.org/) that GPR162 is abundant in the plasma membrane and mitochondrial membrane, with a smaller amount in the nucleus and extracellular space. And STING, as an endoplasmic reticulum transmembrane protein, although it is mostly present on the endoplasmic reticulum membrane, is also found in plasma membranes, mitochondria, and nuclei. This hypothesis was supported by our confocal results in A549 and PC9 cells, where GPR162 was shown to be co-localized with STING (Fig. 1g, h). Following that, we confirmed the location of STING and GPR162 by mitochondrial and endoplasmic reticulum markers, respectively, and the results were consistent with our expectations (Supplementary Fig. 2d). To identify the particular interaction sites of the two, we built locations with a high mutation frequency of GPR162 and STING in lung cancer (Supplementary Tables 2–3). In addition, the immunoprecipitation results showed that the STING-R281L mutant failed to interact with GPR162 (Fig. 1i, j). Furthermore, we performed RNA sequencing (RNA-seq) on GPR162 overexpressed cells and control cells, DESq2 packages, and Gene Set Enrichment Analysis (GSEA) were used for the KEGG (Kyoto Encyclopedia of Genes and Genomes) pathway enrichment analyses were performed. The type I interferon system-pathway was not significantly enriched in GPR162 overexpressed group (Supplementary Fig. 2b, c). Based on these findings, we infer that GPR162 and STING interact directly in the mitochondria and endoplasmic reticulum, but that this connection does not affect downstream transcription.

### GPR162 activates the STING signaling pathway independent of cGAS

**Fig. 2:**
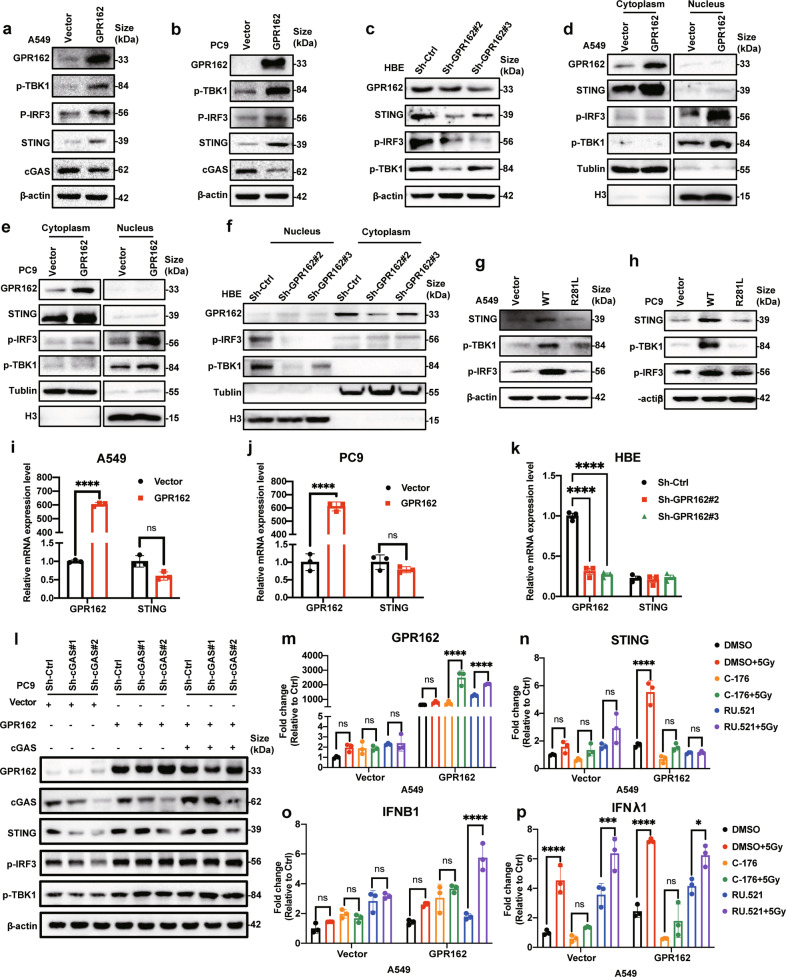
GPR162 activates the STING signaling pathway independent of cGAS. **a**–**c** STING, p-TBK1, p-IRF3, and cGAS expression levels were determined by western-blot analysis in A549 (**a**), PC9 (**b**), and HBE (**c**) cells following GPR162 overexpression or depletion. **d**, **f** STING, p-TBK1, and p-IRF3 expression levels were determined by western-blot analysis in nuclear and cytosolic fractions generated from A549 (**d**), PC9 (**e**), and HBE (**f**) cells following GPR162 overexpression or depletion. **g**–**h** Western-blot analysis was used to evaluate the protein levels of GPR162 and STING-related genes following STING R281L mutation in A549 (**g**) and PC9 (**h**) cells. **i**–**k** qPCR analyses of STING in A549 (**i**), PC9 (**j**), and HBE (**k**) cells after overexpressing or depletion GPR162. **l** The protein levels of STING and its downstream associated components were detected by western-blot in cGAS knockdown PC9 cells following GPR162 overexpression and exogenously introduced cGAS. **m**–**p** qPCR analysis of GPR162 (**m**), STING (**n**), IFNB1 (**o**), and IFNλ1 (**p**) in A549 cells treated with IR by cGAS and STING inhibitors after overexpressing GPR162. (*p < 0.05, ***p < 0.001, ****p < 0.0001)

It is commonly recognized that cGAS-STING is a critical initial immune signaling pathway that plays a vital role in cancer.^3,24,25^ STING and downstream molecules’ mRNA and protein expression levels were observed in liver cancer and lung cancer cell lines overexpressing GPR162. Western blot analysis revealed that after GPR162 overexpression, the protein levels of STING, p-IRF3, and p-TBK1 increased significantly (Fig. 2a, b, Supplementary Fig. 3a, b). On the contrary, after knocking down GPR162, the protein expression of STING, p-IRF3, and p-TBK1 decreased (Fig. 2c). We next isolated cytoplasmic nucleoprotein and measured p-TBK1 and p-IRF3 protein levels because STING activation could activate TBK1 and drive the phosphorylation of transcription factor IRF3 into the nucleus. The results showed that GPR162 can activate STING and further activate downstream molecules (Fig. 2d–f, Supplementary Fig. 3c, d). The mRNA level of STING was detected in GPR162 overexpressed and knockdown liver and lung cancer cell lines, indicating that GPR162 may regulate STING via posttranslational modification rather than transcriptional regulation (Fig. 2i–k, Supplementary Fig. 3e–g). Then we measured STING and downstream protein levels in STING-R281L mutant A549 and PC9 cells and discovered that STING-R281L could indeed inhibit STING and downstream molecule expression (Fig. 2g, h). However, we were surprised to find that overexpression of GPR162 significantly down-regulated the expression level of cGAS protein in liver cancer and lung cancer cells, whereas the cGAS inhibitor (Ru.521) did not affect the transcription level of signaling molecules downstream of STING, while the STING inhibitor (C-176) could significantly inhibit the transcription of downstream molecules (Fig. 2m–p, Supplementary Fig. 3h–q). To prove that GPR162 stimulates STING signaling pathways independent of cGAS, GPR162 was overexpressed in cGAS knockdown cells and exogenously introduced cGAS. We found that the expression of STING, p-TBK1, and p-IRF3 were decreased upon cGAS knockdown, while the protein levels were rescued after GPR162 overexpression in cGAS knockdown cells. And the effect of rescue was not affected by exogenously introduced cGAS (Fig. 2l). These findings show that the STING signal pathway activated by GPR162 may be independent of cGAS.

### GPR162 is involved in the DNA damage pathway and GPR162 overexpression made the cells more sensitive to DNA damage

**Fig. 3:**
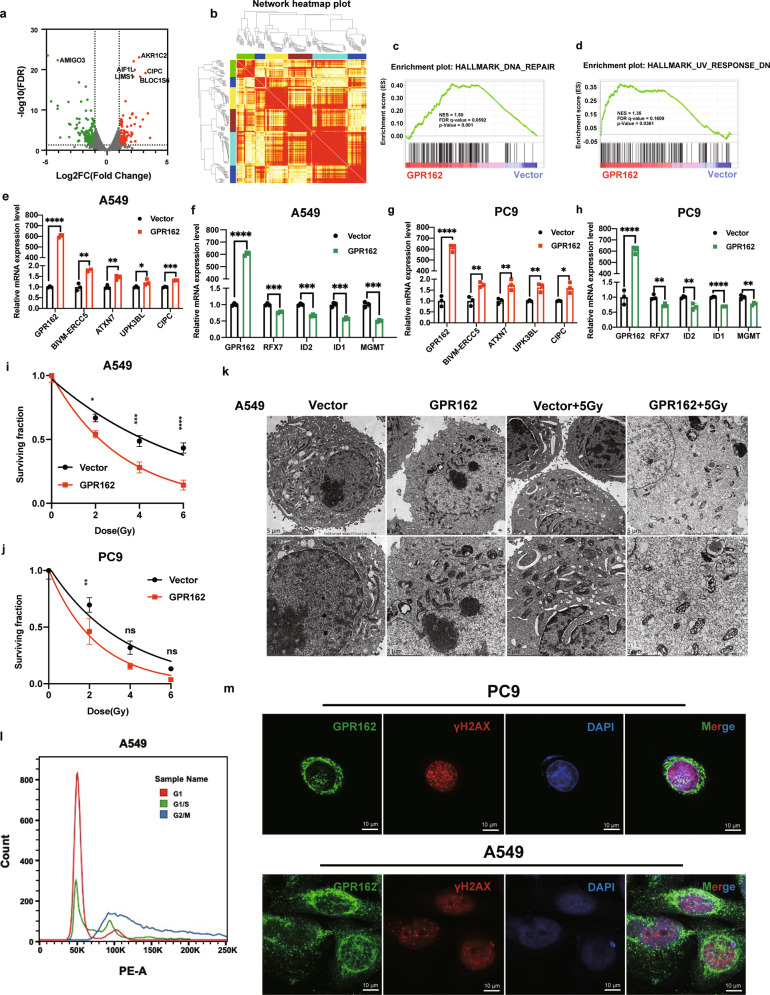
GPR162 is involved in the DNA damage pathway and GPR162 overexpression made the cells more sensitive to DNA damage. **a** Volcanic map was used to analyze transcriptome differential genes in GPR162 overexpressed cells. **b** The correlation coefficient is presented as a heatmap between modules, different color modules represent different data sets. Weighted correlation network analysis (WGCNA) found that brown and aqua blue modules were significantly associated with the phenotype. **c**, **d** GSEA of the whole transcriptome data in GPR162 overexpressed cells were enriched in DNA repair (**c**) and UV response pathway (**d**). **e**–**h** The mRNA levels of DNA damage response-related genes as indicated were examined by RT-qPCR assays in A549 (**e**, **f**) and PC9 (**g**, **h**) cells. **i**, **j** To determine the colony formation ability of A549 (**i**) and PC9 (**j**) cells that were stably overexpressing GPR162, a colony formation assays after radiation on plates at several timepoints. **k** The alterations in the intracellular submicron structure after irradiation in A549 cells with stably overexpressing GPR162 were observed using transmission electron microscopy (TEM). **l** The A549 cell cycle was synchronized in the G1 phase, G1/S phase, and G2/M phase by flow cytometry. **m** Anti-GPR162, anti-γH2AX antibodies, and DAPI were used to label A549 and PC9 cells in confocal microscopy. Scale bar, 10 μm. (*p < 0.05, **p < 0.01, ***p < 0.001, ****p < 0.0001)

To further address how GPR162 triggers the STING signaling pathway, we perform RNA transcriptomics sequencing on overexpressed GPR162 and control A549 cells. We discovered 875 differentially expressed genes (DEGs) (Fig. 3a, Supplementary Fig. 4a, b). Gene Set Enrichment Analysis (GSEA) was used for the KEGG (Kyoto Encyclopedia of Genes and Genomes) pathway enrichment analyses were performed. DNA damage, response to UV-C, and bubble DNA binding were significantly enriched in GPR162 overexpressed group (Fig. 3b–d, supplementary Fig. 4c, d).

Consistent with these results, RT-qPCR revealed that in upregulated DEGs, mRNAs levels of DNA damage response-related genes including BIVM-ERCC5, ATXN7, UPK3BL1, CIPC, B3GAT2, BLOC1S6, AKR1C2, PLA2G4B, AIF1L, MEST, and LIMS1 were upregulated in GPR162 overexpressed group (Fig. 3e–h). This was also confirmed in the down-regulated DEGs (Supplementary Fig. 4j, k).

GPR162 plays a crucial part in the DNA damage pathway, according to RNA-seq and RT-qPCR findings. To investigate whether GPR162 can regulate the DNA damage repair pathway, we analyzed the effect of GPR162 on DNA damage by clone formation assays for irradiation at several time points. The results showed that the cell proliferation after irradiation in GPR162 overexpressed group was considerably lower than the control group, implying that overexpression GPR162 is more sensitive to DNA damage response (Fig. 3i, j, Supplementary Fig. 4n). The same conclusion can be obtained in transmission electron microscopy (TEM), the images showed that the cells in GPR162 overexpressed group suffered more obvious damage after radiotherapy, with a reduced number of mitochondria, swelling of the outer compartment, ridge thickening, endoplasmic reticulum expansion, increased number of nuclear pores, and almost irreversible death trend (Fig. 3k). Interestingly, we found that the fluorescence intensity of γH2AX increased after radiation in GPR162 overexpressed A549 cells (Supplementary Fig. 4m). It is well known that in the late mitotic (M) phase of the cell cycle, with the activity of spindle microtubules, the centromeres split longitudinally, and chromatids unscrew, the cell morphological structure changes rapidly, which is closely related to the periodic changes of various biochemical and nuclear physiology in the cell.^26,27^ Therefore, we used cell synchronization assays to block the cells in the M phase and observed through immunofluorescence that the nuclear entry of GPR162 was significantly increased, and there was a small amount of co-localization with γH2AX (Fig. 3l, m). Therefore, we speculated that GPR162 entered the nucleus in large quantities during the M phase and directly participated in the DNA damage process. These findings suggest that the effect of GPR162 on tumorigenesis is related to DNA damage mechanisms.

### GPR162 promotes DNA damage response in STING-dependent pathways

**Fig. 4:**
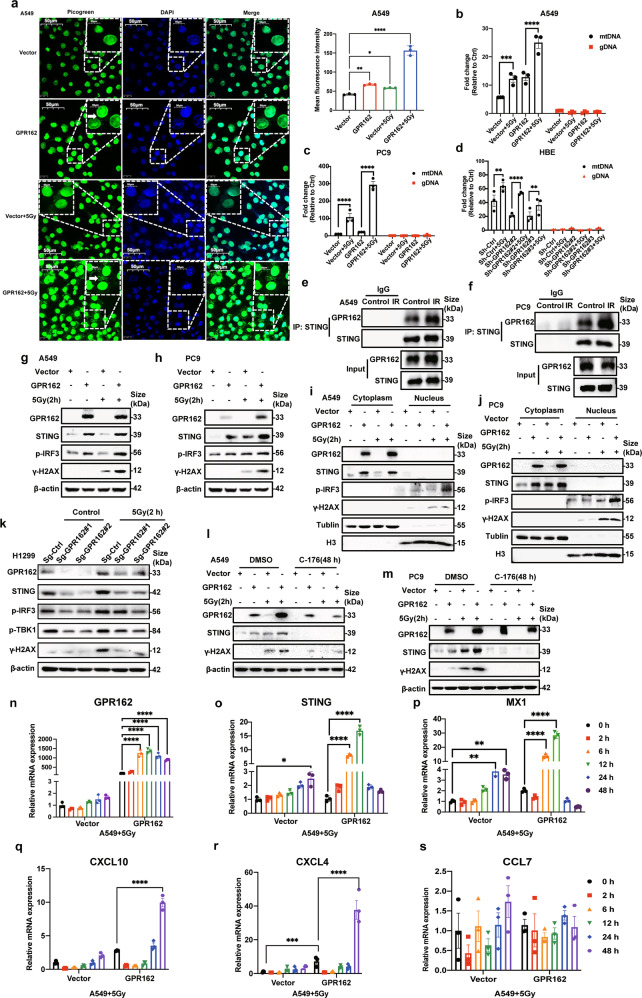
GPR162 promotes DNA damage response in STING-dependent pathway. **a** DNA was identified using the PicoGreen fluorescence dye that specifically binds dsDNA in A549 cells treated with or without IR, as indicated. The cytosolic DNA is indicated by arrows. Each mean fluorescence intensity of PicoGreen was calculated using ImageJ from three different areas. **b**–**d** qPCR analysis of cytosolic DNA with and without IR for 6 h treatment in A549 (**b**), PC9 (**c**), and HBE (**d**) cells after overexpressing or depletion of GPR162. **e**, **f** IP assays were used to analyze the interaction between GPR162 and STING after radiotherapy for 2 h on A549 (**e**) and PC9 (**f**) cells. **g**, **h** After 2 h of IR induction, the protein levels of GPR162, STING, p-IRF3, and γH2AX in A549 (**g**) and PC9 (**h**) cells overexpressing GPR162 were detected by western blot. **i**, **j** GPR162, STING, p-TBK1, and p-IRF3 protein levels in the nuclear and cytoplasmic components of A549 (**i**) and PC9 (**j**) cells overexpressing GPR162 for 2 h after IR induction was detected by western blot. **k** After 2 h of IR induction, the protein levels of GPR162, STING, p-IRF3, and γH2AX in H1299 cells with GPR162 knockout were detected by western blot. **l**, **m** After 2 h of irradiation, the protein expressions of GPR162, STING, p-IRF3, and γH2AX were detected by western-blot in A549 (**l**) and PC9 (**m**) cells overexpressing GPR162. **n**–**s** qPCR analysis of GPR162 (**n**), STING (**o**), MX1 (**p**), CXCL10 (**q**), CXCL4 (**r**), and CCL7 (**s**) mRNA in A549 cells after IR(5 Gy) at different times. (*p < 0.05, **p < 0.01, ****p < 0.0001)

Malignant tumors are frequently associated with the development of chromatin fragments and micronuclei in the cytoplasm, and cancer cells have far more DNA leakage than normal cells.^28,29^ Therefore, the probability of the cGAS-STING signaling pathway being activated in cancer cells is greatly increased. To determine whether the DNA damage repair pathway involved in GPR162 occurs through the activation of the cGAS-STING pathway, we tested the changes in the cytoplasmic DNA distribution of cells in the control group and the GPR162 overexpressed group after radiotherapy at the cellular and molecular levels. We use PicoGreen fuel to measure cytosolic DNA, which is a widely used fluorescent stain that can selectively bind to double-stranded DNA.^25^ We can find that after radiation, the cytosolic DNA positivity percentage was significantly higher in GPR162 overexpressed A549 and PC9 cells than that of the control group and the average fluorescence intensity of the overexpression group was also significantly higher than that of the control group after radiotherapy (Fig. 4a, Supplementary Fig. 5a). At the same time, we also verified the molecular level of mitochondrial DNA after we extracted it (Fig. 4b–d, Supplementary Fig. 5b, c). Meanwhile, we measured the level of mitochondrial DNA in PC9 cells overexpressing GPR162 after radiotherapy at different time periods. The findings reveal that the cytoplasmic DNA continued to increase within 2–24 h after irradiation and attenuated at 48 h in the control group. However, in the overexpression group, cytosolic DNA significantly increased and reached the peak value within 2–6 h after irradiation, and the level of mitochondrial DNA was significantly higher than that of the control group, consequently, the time point of 6 h was selected for our research (Supplementary Fig. 5d). These findings suggest that overexpression of GPR162 can result in the release of DNA from the nucleus to the cytoplasm and that IR promotes this process.

To determine whether the cytoplasmic DNA observed in cells after GPR162 overexpressed activated STING, we used western blot analysis and discovered that after IR treatment, the protein levels of STING, p-IRF3, and p-TBK1 in the GPR162 overexpressed group were significantly higher than the control group (Fig. 4g–j, supplementary Fig. 5k–n). While Dox can induce p53-mediated DNA damage, we investigated the transcription levels of STING and type I interferon-related molecules in A549 and PC9 cells after 18 h of Dox treatment and discovered that both the STING and type I interferon systems were activated (supplementary Fig. 5e, f). It is reported that cytoplasmic DNA accumulation is caused by nuclear DNA damage, we also detected phosphorylation of γH2AX, and the results showed that GPR162 overexpression was more clearly damaged after radiotherapy.^30^ Following that, we measured the protein levels of STING and its downstream associated components, as well as the phosphorylation of γH2AX, in H1299 cells with GPR162 deletion. The protein levels of STING and its downstream molecular proteins were down-regulated following GPR162 deletion, as were the protein levels of phosphorylated γH2AX after radiation (Fig. 4k). Furthermore, protein immunoprecipitation (IP) studies reveal that the protein interactions between GPR162 and STING were strengthened after 2 h of IR and 18 h after Dox induction (Fig. 4e, f, Supplementary Fig. 5i, j), whereas the contact between GPR162 and STING was decreased after 6 h of radiotherapy (supplementary Fig. 5g, h). Consistent with western-blot results, the transcription level of STING, MX1, and IRF7 were upregulated in GPR162 overexpressed group without radiation, while the upregulation was much more significant after radiation therapy. IRF3, a key transcriptional regulator of type 1 interferon-dependent immune responses,^31,32^ binds to an interferon-stimulated response element (ISRE) in the promoters of type I IFN genes (IFN-alpha and IFN-beta) and IFN-stimulated genes (ISG) to regulate transcription,^33,34^ mainly affects the expression of chemokines including CXCL10 and CXCL4 in DNA damage response.^35^ Therefore, we measured the mRNA expression of CXCL10 and CXCL4 in A549 and PC9 cells overexpressing GPR162. Compared with the control group, the mRNA levels of CXCL10 and CXCL4 were upregulated in the GPR162 overexpression group without radiation therapy, while the upregulation was much more significant after radiotherapy (Fig. 4n–s, Supplementary Fig. 6). Finally, we treated GPR162 overexpressed A549 and PC9 cell lines with STING inhibitors (C-176) for 24 h, then irradiated the cells with radiation for 2 h. We discovered that GPR162 could accelerate DNA damage, while STING inhibitor (C-176) could alleviate DNA damage (Fig. 4l, m). This meant that the DNA damage response induced by GPR162 was dependent on the STING pathway.

### GPR162 is lowly expressed in multiple types of cancer

**Fig. 5:**
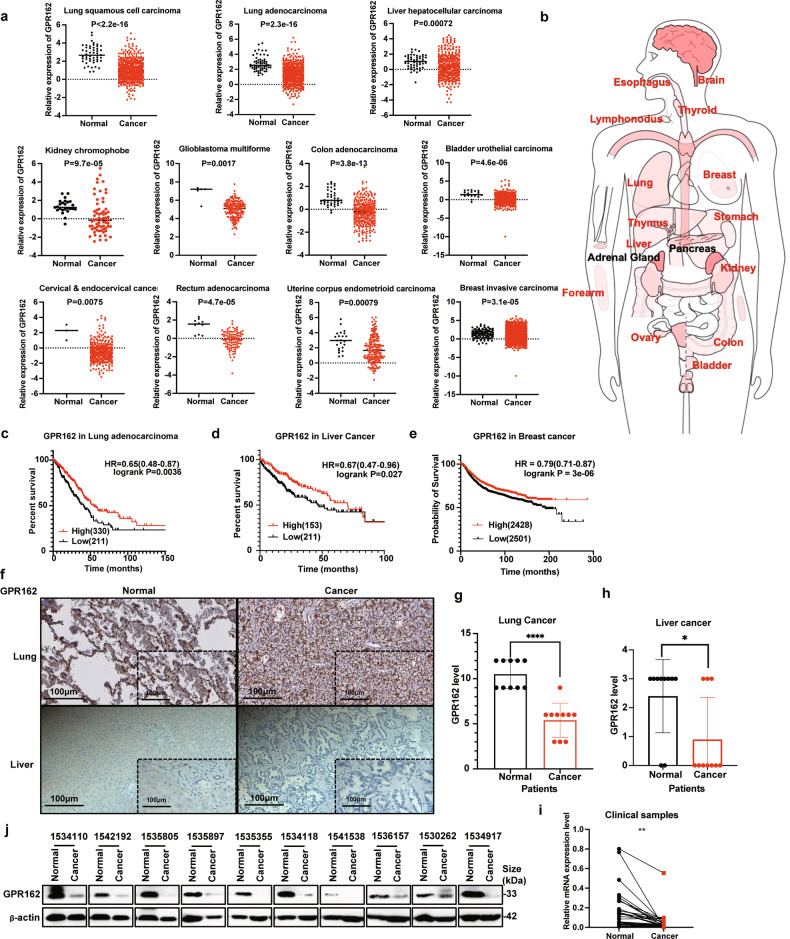
GPR162 is lowly expressed in multiple types of cancer. **a** TCGA analysis of GPR162 mRNA expression in lung squamous cell carcinoma, lung adenocarcinoma, liver hepatocellular carcinoma, kidney chromophobe, glioblastoma multiforme, colon adenocarcinoma, bladder urothelial carcinoma, cervical & endocervical cancer, rectum adenocarcinoma, uterine, corpus endometrioid carcinoma, and breast invasive carcinoma compared to each normal sample. Each dot represents a sample. **b** Human models showed low expression of GPR162 in most solid tumors. Body image from *Gene Expression Profiling Interactive Analysis*. **c**–**e** In lung adenocarcinoma (**c**), liver cancer (**d**), and breast cancer (**e**), Kaplan–Meier curves demonstrate overall survival rates related to GPR162 expression. The log-rank test was used to evaluate the results. **f** IHC analysis of GPR162 expression level in lung adenocarcinoma and liver cancer clinical samples. **g**, **h** GPR162 expression levels are higher in lung adenocarcinoma (**g**) and liver cancer (**h**) tissues than in normal tissues, according to IHC scores. **i** mRNA expression of GPR162 was found to be lower in 30 paired lung cancer tissue samples compared to neighboring normal lung tissue samples by qPCR. Each dot represents a different sample. **j** The protein level of GPR162 was found to be lower in 10 paired lung cancer tissue samples compared to neighboring normal lung tissue samples from western-blot analysis. (*p < 0.05, **p < 0.01, ****p < 0.0001)

To further investigate the significance of GPR162 in clinical, we examined GPR162 mRNA levels in a range of cancer patients from the TCGA database. Surprisingly, we discovered that GPR162 expression was lower in almost all solid tumors than in normal tissues, Figure B from the website (http://gepia.cancer-pku.cn/) shows the expression and distribution of GPR162 in tumors tissues (Fig. 5a, b, Supplementary Fig. 7a, b). Kaplan–Meier analysis evaluated the survival rate of patients with liver cancer, lung adenocarcinoma, and breast cancer, and found that patients with high GPR162 expression had a better prognosis (Fig. 5c–e). The findings revealed that high GPR162 expression correlates with a high survival rate. In addition, we examined clinical tissue samples from individuals with lung cancer and liver cancer using immunohistochemistry. We discovered that the immunohistochemistry score of GPR162 in lung cancer and liver cancer tissues was lower than that of neighboring normal tissues when compared to normal tissues (Fig. 5f–h). In addition, we detected the protein and mRNA levels of GPR162 in lung adenocarcinoma tissues and normal tissues adjacent to cancer and found that GPR162 was expressed higher in normal tissues (Fig. 5i, j). Meanwhile, GPR162 protein and mRNA levels were measured in lung cancer cell lines and normal lung tissue cells, it was shown that the expression of GPR162 in normal cells was much higher than those in lung cancer cell lines (Supplementary Fig. 7c, d). These results indicate that GPR162 can reduce the clinical response of patients in clinical tumor models.

### GPR162 overexpression slowed cell proliferation, colony formation, transwell formation, and tumor development

**Fig. 6:**
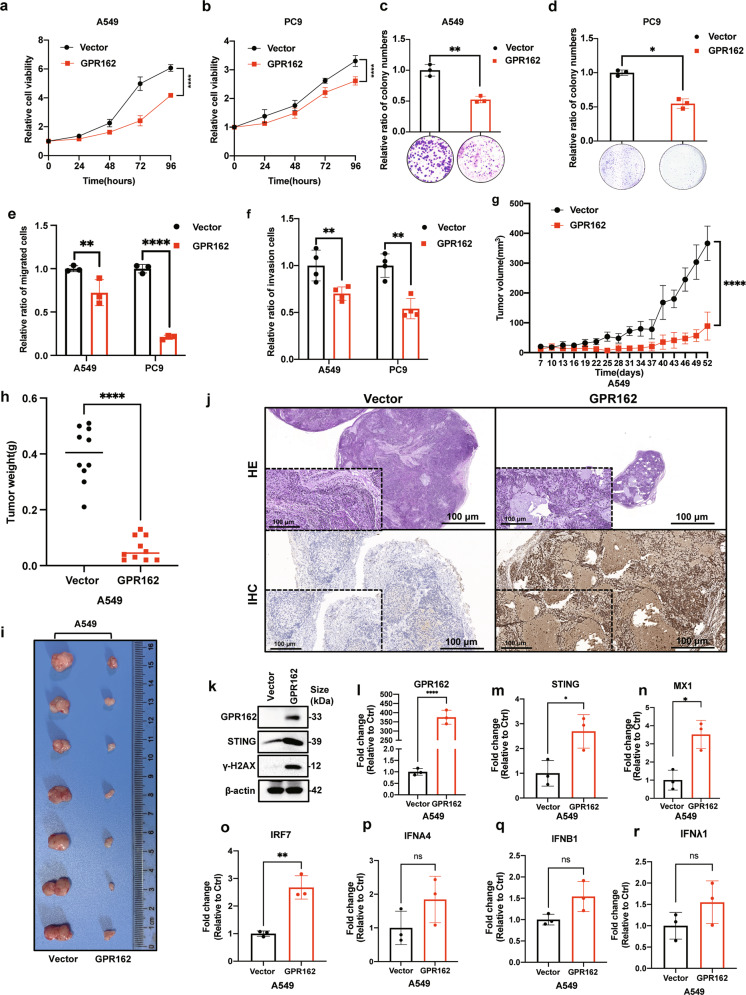
GPR162 overexpression slowed cell proliferation, colony formation, transwell migration and invasion, and tumor development. **a**, **b** Cell viability was determined using the CCK8 assay in A549 (**a**) and PC9 (**b**) cells that were stably overexpressing GPR162. **c**, **d** A colony formation experiment on plates was used to assess the ability of A549 (**c**) and PC9 (**d**) cells overexpressing GPR162 to form colonies. **e**, **f** The migration and invasion of A549 and PC9 cells overexpressing GPR162 were detected using a transwell test. **g**, **i** To investigate the capacity of A549 cells with stable GPR162 overexpression to develop tumors (*n* = 10 mice per group), a tumor growth xenograft model was established. Tumor formation was tracked at the indicated times (**g**), weight (**h**), and image (**i**). **j** The tumor tissue of the xenograft model was evaluated by HE and IHC. **k** GPR162, STING, and γH2AX expression levels in tumor tissue were determined by western blot analysis. **l**–**r** qPCR analyses of GPR162 (**l**), STING (**m**), MX1 (**n**), IRF7 (**o**), IFNA4 (**p**), IFNB1 (**q**), and IFNλ1 (**r**) in tumor tissue. (*p < 0.05, **p < 0.01, ****p < 0.0001)

To reveal the physiological significance of GPR162 in lung cancer, we constructed cell lines stably overexpressing GPR162 in A549 and PC9 cells. As expected, the cell proliferation was much lower in GPR162 overexpressed cells than that of control cells (Fig. 6a, b). Furthermore, overexpression of GPR162 dramatically decreased cell colony formation, migration, and invasion (Fig. 6c–f, Supplementary Fig. 8a, b).

A xenograft model experiment was used to further investigate the influence of GPR162 on tumor development in vivo. The injection of A549 cells overexpressing GPR162 can drastically Inhibit tumor growth, volume, and weight when compared to the injection of control cells, while the overall body weight of the mice remains unchanged (Fig. 6g–i, Supplementary Fig. 8c). At the same time, HE and IHC staining were performed on subcutaneous tumor tissues of nude mice (Fig. 6j). The protein and mRNA levels of STING-related molecules from subcutaneous tumor tissues of nude mice were detected using western-blot and RT-qPCR assays (Fig. 6k–r). We found that the protein level of STING increased significantly after overexpression, but the mRNA level had no significance, which was in line with expectations. Finally, these findings show that GPR162 overexpression is associated with cell proliferation, colony formation, migration and invasion, and tumor development, as well as having a strong negative effect on tumor progression.

### GPR162 knockout enhances cell proliferation, colony formation, transwell formation, and tumor development

**Fig. 7:**
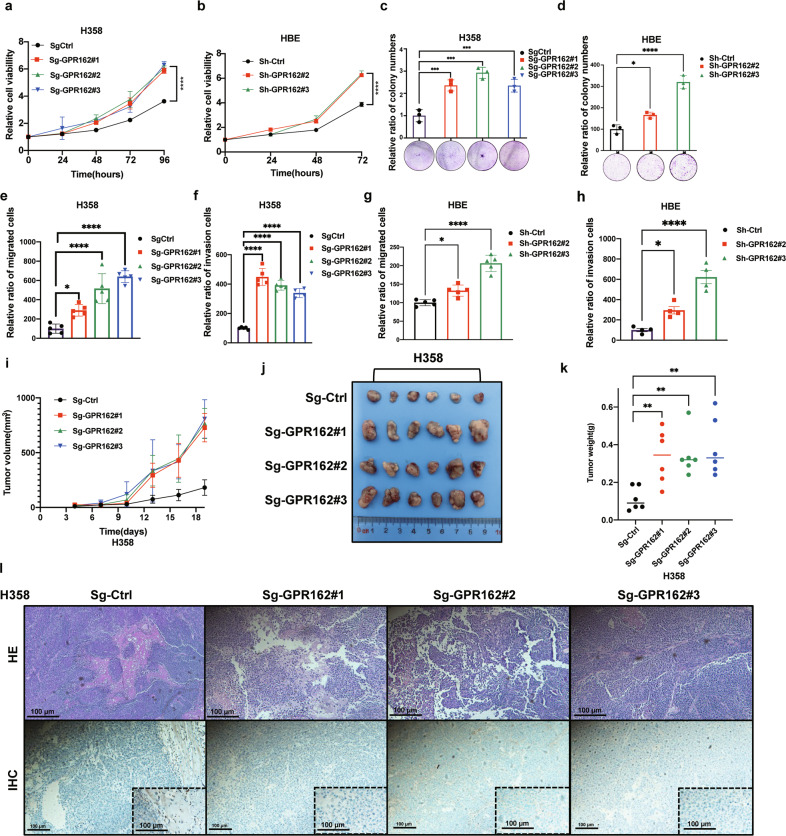
GPR162 knockout enhances cell proliferation, colony formation, transwell migration and invasion, and tumor development. **a**, **b** Cell viability in H358 (**a**) and HBE (**b**) cells with stably knockout and knockdown GPR162 was assessed using the CCK8 assay. **c**, **d** A colony formation assay in plates was used to determine the ability of H358 (**c**) and HBE (**d**) cells with stably knockout and knockdown GPR162 to form colonies. **e**–**h** GPR162 migration and invasion were detected using a transwell assay in H358 (**e**, **f**) and HBE (**g**, **h**) cells with stable deletion and knockdown of GPR162. **i**–**k** To assess the capacity of H358 cells with stable GPR162 deletion to produce tumors (*n* = 10 mice per group), a tumor growth xenograft model was established. Tumor formation was tracked at the times indicated (**i**), pictures (**j**) and weight (**k**) are shown. **l** HE and IHC staining of tumor tissue from the xenograft model. (**p < 0.01, ***p < 0.001, ****p < 0.0001)

Next, we stably knocked out GPR162 in the H358 cell line and knocked down GPR162 in the HBE cell line, also detecting the physiological effects of GPR162 depletion on lung cancer. The results showed that the cell proliferation of GPR162 depletion cells was significantly higher than that of control cells (Fig. 7a, b), and the depletion of the GPR162 gene promoted the colony-forming ability, migration, and invasion ability of cells (Fig. 7c–h, Supplementary Fig. 9a–d).

H358 cells were injected into nude mice to evaluate tumor development in vivo. GPR162 deletion resulted in a considerable rise in tumor growth, volume, and weight, according to our findings (Fig. 7i–k). The body weight of mice in the four groups of samples, however, did not differ significantly (Supplementary Fig. 9e). In general, these results indicate that the absence of GPR162 significantly promotes the occurrence and development of tumors.

### GPR162 promotes radiation-induced antitumor effects by activating the STING signaling pathway

**Fig. 8:**
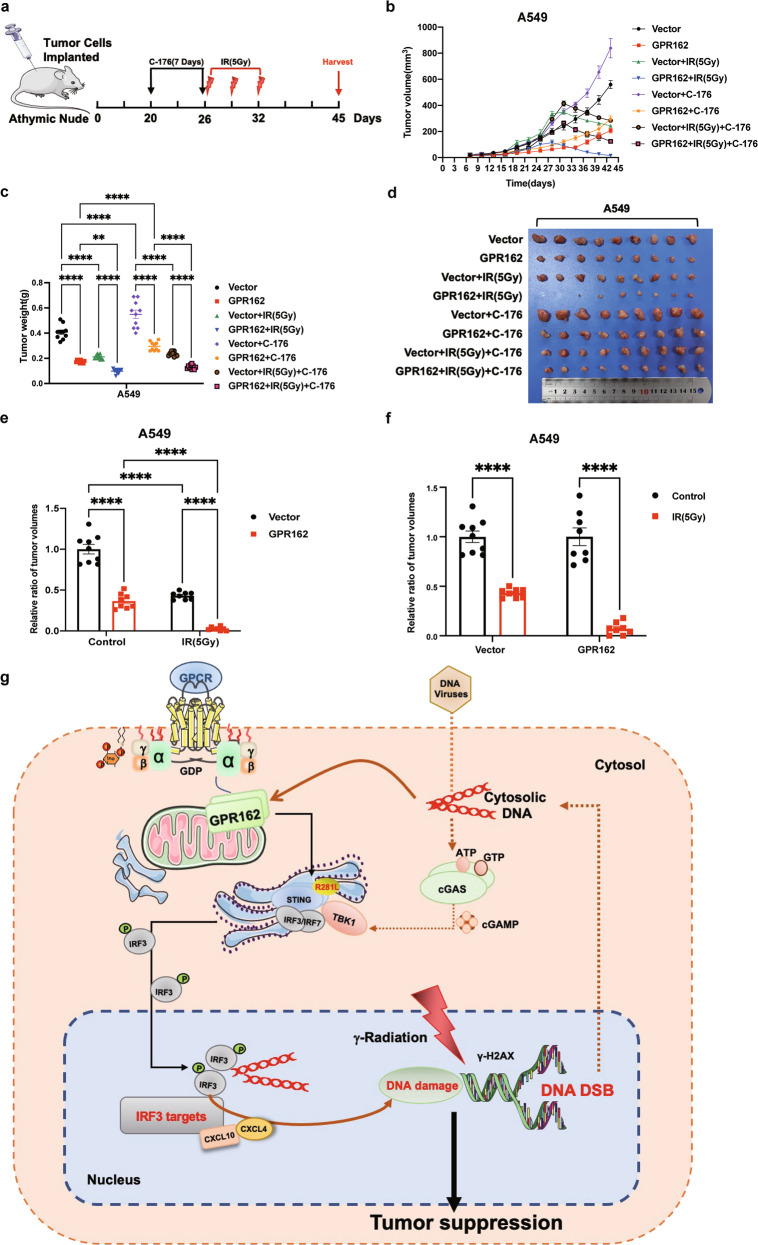
GPR162 increases the anticancer impact of radioimmunotherapy by stimulating the STING signaling pathway. **a** Treatment schedule of IR and C-176. **b**–**d** Subcutaneous tumors of A549 cells respond to the specified therapies. Each group has ten mice. Tumor formation was tracked at the times indicated (**b**), weight (**c**), and pictures (**d**) are shown. **e**, **f** The tumor volume of the control group and the overexpression group. **g** Schematic model of radio-induced GPR162-STING-DNA damage in cancer: GPR162, a novel tumor suppressor, can promote the DNA damage response induced by radiotherapy to activate STING, and further activate STING to induce DNA damage of tumor cells and inhibit the occurrence and development of tumors

Previous studies have confirmed that GPR162 can promote STING-related pathway-dependent DNA damage responses, thus we want to see if the GPR162-STING-DNA damage axis can be employed as a transformation strategy to boost the anti-tumor effect of radiation. To solve this problem, we used a competitive inhibitor of STING (C-176) in the nude mice of the subcutaneous xenograft model. To determine the tumor inhibitory effects of radiotherapy and STING on GPR162, different groups were used to treat A549 tumor-bearing mice (Fig. 8a). When the tumor reaches about 200 mm^3^, radiotherapy is performed. The STING inhibitor was injected intraperitoneally every day from one week before the radiation for 7 consecutive days, and the tumor volume was measured at the same time. The results showed that irradiation can significantly inhibit tumor growth, and the effect was more significant in the GPR162 overexpression group (Fig. 8a–d), although there was no difference in the body weight of the mice (Supplementary Fig. 9f). The tumor volume of the control group was reduced by around 55% following radiotherapy, whereas the volume of the overexpression group was reduced by about 85%, implying that the overexpression GPR162 group was more susceptible to radiotherapy (Fig. 8e, f). Furthermore, STING inhibitors can greatly reduce the anti-tumor impact of radiotherapy, implying that GPR162 increases the radiotherapy sensitivity of tumors by activating the STING signaling pathway.

In conclusion, GPR162 can be used as a novel tumor suppressor to promote the activation of STING in the DNA damage response induced by radiotherapy, and then activate STING to induce DNA damage of tumor cells, thus inhibiting the occurrence and development of tumors (Fig. 8g). The above results indicated that the GPR162-STING axis enhances the anti-tumor effect induced by radiotherapy, which may provide a new approach to cancer treatment.

## Discussion

Radiotherapy has become one of the common treatment strategies for patients with advanced tumors,^36^ which mainly depends on the regulation of radiotherapy on the tumor immune microenvironment,^37–39^ and the inhibition of the immune microenvironment caused by DNA damage repair (DDR) pathway activation induced by IR,^40^ thus affecting the anti-tumor effect of radiation immunotherapy.^41,42^ However, the poor radiotherapy effect caused by IR resistance of tumor cells is still one of the main reasons for tumor recurrence and metastasis. Therefore, there is a critical need to enhance therapeutic options for tumor radiation tolerance. In addition, it is not clear whether activation of the double-stranded breaks (DSBs) repair pathway enhances the efficacy of radioimmunotherapy. Our findings show that GPR162 increases the radiation-induced DNA damage response and is involved in the activation of the type I interferon system.

DDR is linked to inflammatory signal transduction and plays a critical role in anti-tumor immunity, according to recent research.^43^ Radiotherapy can induce DNA damage in the nucleus and promote the entry of broken DNA fragments into the cytoplasm to activate STING signaling pathways.^2,35^ In our study, we found that GPR162 can promote RT-induced DDR and activate the STING-TBK1-IRF3 innate immune pathway, leading to a significant increase in the transcription level of proinflammatory cytokines, such as CXCL10, and CXCL4, further promoting DNA damage. Studies have reported that these chemokines induce the activation and function of cytotoxic T lymphocytes,^35^ CXCL10, a 10 kDa protein, which is categorized functionally as a Th1-chemokine. It binds to the receptor CXCR3 and regulates immune responses through the activation and recruitment of leukocytes, such as T cells, eosinophils, and monocytes,^44^ is strongly induced by IFN-γ as well as by IFN-α/β and weakly by TNFα. In vitro, CXCL10 can also be induced by NF-kB and has been shown to have an early role in hypoxia-induced inflammation. Activation of IFN-regulatory factor 3, toll-like receptors, retinoic acid-inducible gene (RIG)-I, and melanoma differentiation-associated gene (MDA)-5 work in synergy with IFNs for CXCL10 induction.^45^ CXCL4 is associated with macrophages, affecting the differentiation of monocytes and inducing specific macrophage phenotypes.^46^ Through bioinformatics, we predicted the relationship between GPR162 and immunity, and also found that GPR162 was significantly positively correlated with Th1 and macrophages in lung adenocarcinoma and lung squamous cell carcinoma, which was consistent with previous results. In consideration of these findings, it is extremely interesting how GPR162 activation on ISGs genes and related tumor immunity, which will be explored in our further studies.

Many molecules have been proposed as direct cytoplasmic DNA sensors, including DAI, p204,^47^ IFI204 (IFI16), Toll-like receptors (TLR3, 7, 8, 9), AIM2-like receptors (AIM2, IFI16), RNA polymerase III, DExD/H box nucleic acid helicases (such as RIG-I like receptors (RIG-I, MDA5, LPG2), DDX1, 3, 5, 7, 17, 21, 41, 60, and DHX9, 36).^48,49^ cGAS and the DNA repair protein Mre11 have recently been postulated as direct cytoplasmic DNA sensors that work upstream of STING to generate type I IFN.^50,51^ cGAS has also recently been reported to be independent of cGAS-STING, providing new insights into DNA repair.^52^ Our findings imply that GPR162 can behave as a new molecule that activates STING and generates type I IFN without using the traditional cGAS-STING signaling pathway, and that it can help with the identification of cytoplasmic DNA damaged by DNA damage. This could be a sophisticated and intricate control of IFN production in response to various stimuli.

A growing body of evidence indicates that GPCR is closely related to a wide range of diseases, including genetic, tumor, neurological and reproductive system diseases, which is of great significance for the development of GPCR therapeutics, and many of the current drugs targeting GPCRs have excellent therapeutic effects.^53,54^ For example, orphan receptor GPR124 may be a therapeutic target for central nervous system-related vascular diseases^55^; the GPR171 pathway can reduce anti-tumor immunity by inhibiting T cell activation^56^; Key mechanisms implicated in the escape of uveal melanoma cells from MEK inhibition include GPCR-mediated YAP activation and RTK-driven AKT signaling.^57^ However, until now, the physiological role of many peptides and protein-coupled receptors (GPCRs) exceeding 100 G remains unclear.^58^

Here, we used the TCGA database to analyze the expression and survival curves of GPR162 in a variety of solid tumors, including lung adenocarcinoma, and found that the reduction of GPR162 led to more tumors and reduced survival rates. Combined with in vitro experiments, We discovered that IR dramatically boosted GPR162 protein expression, indicating a potential role for this orphan type of GPCR in tumor irradiation. Importantly, in the nude mouse subcutaneous xenograft model, we observed that subcutaneous tumors of GPR162 overexpressed mice were significantly reduced after radiotherapy.

In conclusion, as a novel tumor inhibitor and radiotherapy sensitizer, GPR162 can promote the entry of DNA damage into the cytoplasm and activate the STING-IFN system to improve cancer radiotherapy.

## Materials and methods

### Cell culture, viruses, stimulation, transfection, and γ-irradiation

In this investigation, the following cell culture conditions were used: A549 (ATCC: CCL-185) cell lines were cultured in 1:1 DME/F12 (HyClone, UT, USA) medium, PC9, HBE (ATCC: CRL-2078), H358 (ATCC: CRL-5807) cell lines were cultured in RPMI1640 (Gibco) medium, and DMEM (Gibco, NY, USA) medium were used to culture Hep3B (HB-8064), HepG2 (HB-8065) and HEK-293T cell lines. Cells were cultured in a cell incubator at 37 °C with 5% CO_2_ and the medium containing 10% (v/v) BCS. All cell lines were obtained from the cell bank of Cancer Institute, Central South University. Vigene Biosciences provided the GPR162 cDNA clones. GPR162 cDNA was inserted into the third generation lentivirus expression PLVX-EF1α-IRES-Puro vector (Catalog No. 631988; Clontech, CA, USA) to construct FLAG-GPR162 overexpression plasmid. The targeted shRNA sequence is derived from GPP Web Portal (https://portals.broadinstitute.org/gpp/public/gene/search), lentivirus expressed PLVX-shRNA1 vector (Catalog No. 632177; Clontech, CA, USA) was inserted into the target plasmid vector for GPR162-shRNA plasmid construction. The sequences of sgRNAs and shRNAs for GPR162 in this paper are listed in Supplementary Table 5. The constructed plasmid was introduced into cells and transfected with Lipofectamine Max, and the colonies with stable expression were screened by puromycin (1 μg/ml). All cell lines and animals were irradiated from the Central Laboratory of Xiangya Hospital.

### Western blot analysis and coimmunoprecipitation (Co-IP) assay

The collected cells were washed three times with 1 × PBS and then lysed on ice in IP lysis buffer containing protease inhibitor cocktail for 1 h. The protein concentration was determined by the BCA method and the system was prepared. Total proteins obtained from cell lysis were isolated using SDS-polyacrylamide gel and then transferred to a polyvinylidene fluoride membrane. Primary antibodies used for Western blot analysis are listed in Supplementary Table 6.

The proteins pre-cleared by magnetic beads were added with target protein antibodies at 4 °C and incubated overnight. After adsorption by magnetic beads, the proteins were denatured and the interaction between proteins was detected by Western blot analysis.

### Real-time quantitative polymerase chain reaction (RT-qPCR)

Total RNA was separated using TRIzol reagent (Takara, Kusatsu, Japan), and the RNA was reverse transcribed into cDNA using the kit (Takara, Kusatsu, Japan). On Bio-rad CFX Connect real-time PCR apparatus, real-time PCR was done. The internal reference for gene expression was β-actin. Supplementary Table 5 lists the primers used in this study.

### Immunofluorescence microscopy

The logarithmic growth cells were planted in a 24-well culture plate with small glass discs 24 h in advance for the adherent growth cells, and the culture plate was removed when the degree of cell fusion reached about 50%. After three washes with 1 × PBS, 1 mL methanol was applied to each well and fixed at 20 degrees for 10 min. And then rinsed with PBS for 5 min twice, blocked with 1% (w/v) BSA in PBS for 30 min. Rinsed with PBS for 5 min three times and incubated with primary antibodies containing 1% (w/v) BSA incubate at 4 °C overnight. After washing with PBS, anti-rabbit IgG Alexa 594 fluorescent secondary antibody or anti-mouse IgM Alexa 488 fluorescent secondary antibody was selected and incubated for 1 h according to the properties of the primary antibody. Finally, DAPI staining was performed, and they were mounted on slides and imaged with Leica TCS SP8 confocal microscope. ER-Tracker^TM^ Blue-White DPX dye (Invitrogen, 374/430–640 nm, E12353) was used for endoplasmic reticulum staining of living cells and was incubated with 500 nM ER-tracker in a cell incubator at 37 °C with 5% CO_2_ for 30 min. MitoTracker^®^ Deep Red FM (Invitrogen, 644–665 nm, M22426) was used for mitochondrial staining of living cells and was incubated with 500 nM ER-Tracker in a cell incubator at 37 °C with 5% CO_2_ for 30 min.

### Transmission electron microscopy (TEM)

A549 cells overexpressing GPR162 were seeded onto 6 cm plates and treated with IR(5Gy) for 6 h. After the cells were digested and collected with trypsin, wash the cells twice with PBS, add 1 mL fixative solution along the wall of the centrifuge tube (the cells should not be scattered), and refrigerate at 4 °C overnight. The image was captured with a transmission electron microscope (Hitachi; HT7700) from Xiangya Hospital’s Department of Pathology.

### Cell synchronization

The double cell cycle blockade analysis was carried out exactly as stated previously.^26^ Treatment with 50 ng/ml Nocodazole for 12 h synchronized A549 cells to mitosis, after which the cells were released in new media for 3.5 h. The M phase is then followed by double blocking. The cells were initially cultured in 2 mM thymidine for 24 h, then released in a fresh medium for 12 h following the first block. The cells were also cultured for 24 h in 2 mM thymidine. Finally, cells were discharged for 12 h into a fresh medium to harvest M phase cells.

### Quantification of cytoplasmic DNA

The cytoplasmic DNA was quantified in the same manner as previously described.^25^ Cells were grown on a 24-well plate with a cover glass for the immunofluorescence microscopy experiment. After IR (5 Gy) irradiation for 6 h, the cells were washed twice in cold PBS and fixed for 10 min in cold methanol at −20 °C. After three PBS washes, cells were blocked for 1 h with 1% BSA in PBS and stained for 1 h with Pico488 dsDNA quantification reagent(Lumiprobe, USA, 42010). The cover glass was photographed with a Leica TCS SP8 confocal microscope after being cleaned three times with PBS and mounted on white microscope slides with ProlongTM Diamond Antifade Mountant with DAPI.

The cytosolic DNA quantification assay is performed as follows, gDNA was extracted by boiling method and cytoplasmic DNA was extracted by digestion method. After trypsin digestion, the cells were washed twice with PBS, adding 100 μL 50 μM NaOH, boiling at 98 °C for 15 min, adding 10 μL 1 M Tris-HCl PH 8.0 to neutralize NaOH in a lysis solution, swirling for 10 s, and standing at 4 °C for 30 s. At 13,000 × *g*/10 min, the supernatant was removed for crude extraction of gDNA, 10 μL 25 mg/mL Protein K was added at 60 °C and stood for 45 min. gDNA was purified and concentrated from the DNA Chem concentrator (ZYMO Research cat.no 4033). Add 200 μL Cytosolic DNA extract buffer (150 mM NaCl 500 mM HEPES, 250 g/mL digestion), stand on ice for 15 min, centrifuge 13,000 × *g* at 4 °C for 2 min, absorb supernatant, add 10 μL 25 mg/mL Protein K, 60 °C for 45 min and then Cytosolic DNA was obtained by DNA Chem concentrator (ZYMO Research cat.no 4033). The obtained cytosolic DNA was quantified by qPCR, gDNA was used as input, Poly primers were used as gDNA-specific primers, ND1 was cytosolic DNA-specific primers and the expression level of ND1 was used as cytosolic DNA reference.

### Separation of the cytoplasmic nucleus

The cytoplasmic nucleus was separated as previously described.^59^ Adherent cells were collected and suspended in 10 cm dishes by adding 200 μL buffer A (10 mM HEPES, pH 7.9, 10 mM KCl, added with protease inhibitor). The cells were put on ice for 15 min, then 10 percent Nonidet P-40 was added to 0.625%, followed by 10 s vortex oscillation to release the cytoplasmic proteins. The cytoplasmic fraction was centrifuged at 10000 g for 30 s at 4 °C. 1 mL buffer A, 14,000 × *g*, 4 °C, 2 min, followed by 50 μL buffer B (buffer A containing 1% SDS).

### Cell proliferation, transwell, and colony formation assays

As previously disclosed, cell proliferation, transwell, and colony formation experiments were carried out.^60^

According to the manufacturer’s instructions, Cell Counting Kit-8 was used in the cell proliferation experiment. First, 1000 cells were plated into each well of a 96-well plate, with 5 sub-wells in each group. The OD450 was determined 2 h after the CCK8 reagent was added.

In the cell transwell test, dilute Matrigel (Becton, Dickinson and Company, USA) at a 1:8 ratio with serum-free culture media. Combine the ingredients and pour 50 L into the chamber. 2 × 10^5^ cells were added to each chamber, and the cells were fixed with methanol and stained with 0.5% crystal violet 48 h later. A light microscope was used to capture the images.

In the cell colony formation experiment, 500 cells were seeded into each well of six-well plates, which were subsequently grown in a cell incubator. Two weeks later, the cells were preserved in methanol and stained with 0.5% crystal violet. A microscope and ImageJ software were used to count the clones (1.47 v, NIH, USA).

### Nude mice and study approval

As previously reported, the xenograft tumor experiment was carried out.^15^ Female SCID mice, 4–6 weeks old, were procured from Hunan SJA Laboratory Animal Co., Ltd. (Changsha, China). Animal studies were carried out with the agreement of Central South University’s Xiangya School of Medicine’s Institutional Animal Care and Use Committee and by legislative regulations and federal standards for animal protection and care. Each mouse’s axilla was injected subcutaneously with GPR162-overexpressing or GPR162-silenced cells, as well as the matching control cells (1 × 10^6^ cells/mouse). After then, tumor volume and mouse weight were monitored every three days until the mice were euthanized at 52 days. Tumors were weighed, fixed in 10% formalin, and then paraffin-embedded or processed for RNA and protein extraction.

### Mice treatments

Female SCID mice, 4–6 weeks old, were procured from Hunan SJA Laboratory Animal Co., Ltd. (Changsha, China). Each mouse’s axilla was injected subcutaneously with GPR162-overexpressing or GPR162-silenced cells, as well as the matching control cells (1 × 10^6^ cells/mouse). The tumor volume was assessed every two days, and C-176 (Selleck, S6575, 5 mg/kg/day) was given intraperitoneally to the mice for seven days. When the tumor reached about 200 mm^3^ (tumor diameter was about 7.25 mm), the mice were irradiated with radiotherapy. The mice were irradiated with 5 Gy three times on the 20, 23, and 26 days. For ethically, mice were sacrificed when the tumor volume reached 1000 mm^3^, about 60 days after the beginning of treatment.

### Histology and immunohistochemistry

Xiangya Hospital’s Department of Pathology confirmed and gave biopsies of lung cancer and related disorders. Previous literature describes the procedure for IHC examination of paraffin slices from lung cancer tissues. Two pathologists from Xiangya Hospital in Changsha, China, used a CX41 microscope (Olympus, Tokyo, Japan) with a DP-72 microscope digital camera system (Olympus, Tokyo, Japan) to record images of the paraffin sections, and differential quantification was conducted by two pathologists from Xiangya Hospital in Changsha, China.

### Statistics

Studies were performed at least three times, except for the nude mice experiments. The data is presented as a mean SD or SEM. Statistical analyses were carried out using the GraphPad Prism 9.0 program. The significance of differences between two groups was determined using the *T*-test, and analysis of variance (ANOVA) was performed to analyze more than two groups. For correlation analysis, the Parsons correlation coefficient was utilized. In the following situation, differences were deemed statistically significant: *p* < 0.05 (*p < 0.05, **p < 0.01, ***p < 0.001, ****p < 0.0001).

### Study approval

The study was authorized by our hospital’s ethics committee. Adenocarcinoma archive data was gathered from the Xiangya Pathologic Anatomy Service’s files. The use of animal models in this work was approved by Central South University’s institutional Animal Care and Use Committee. All of the participating medical facilities’ institutional review boards gave their approval to the study. Before enrollment, all study participants signed a written informed consent form.

## Supplementary information

GPR162 activates STING dependent DNA damage pathway as a novel tumor suppressor and radiation sensitizer

## Data Availability

All data supporting this paper are available from the corresponding authors upon reasonable request. Raw sequencing data have been uploaded to SRA: SUB12060617 (https://dataview.ncbi.nlm.nih.gov/object/PRJNA880571?reviewer=do4bvq1qr9be58hp8551hg9a73).
