# Author Correction: GPR162 activates STING dependent DNA damage pathway as a novel tumor suppressor and radiation sensitizer

**DOI:** 10.1038/s41392-023-01508-2

**Published:** 2023-06-30

**Authors:** Yao Long, Jiaxing Guo, Jielin Chen, Jingyue Sun, Haiyan Wang, Xin Peng, Zuli Wang, WeiWei Lai, Na Liu, Long Shu, Ling Chen, Ying Shi, Desheng Xiao, Shuang Liu, Yongguang Tao

**Affiliations:** 1 Department of Pathology, Key Laboratory of Carcinogenesis and Cancer Invasion(Ministry of Education), Xiangya Hospital, Central South University, Changsha, Hunan 410078 China; 2 NHC Key Laboratory of Carcinogenesis of Ministry of Health (Central South University), Cancer Research Institute, School of Basic Medicine, Central South University, Changsha, Hunan 410078 China; 3 Department of Pathology, Xiangya Hospital, Central South University, Changsha, Hunan 410008 China; 4 Hunan Key Laboratory of Tumor Models and Individualized Medicine, Department of Thoracic Surgery, Second Xiangya Hospital, Central South University, Changsha, China; 5 Hunan International Scientific and Technological Cooperation Base of Brain Tumor Research, Xiangya Hospital, Central South University, Changsha, Hunan 410008 China

**Keywords:** Cancer genetics, Cell biology

Correction to: *Signal Transduction and Targeted Therapy* (2023) 8:48 10.1038/s41392-022-01224-3, Published 1 February 2023

Since the publication of this article, we noticed a minor mistake in the article that needs to be corrected. We have checked the original data; the correct data are provided in this Corrigendum as follows. The key findings of the article are not affected by these corrections.

In the original version of this article^1^, the photomicrograph showing a representative image of A549 cells treated with or without IR in Fig. 4a was incorrect. The image has now been corrected.

**Figure:**
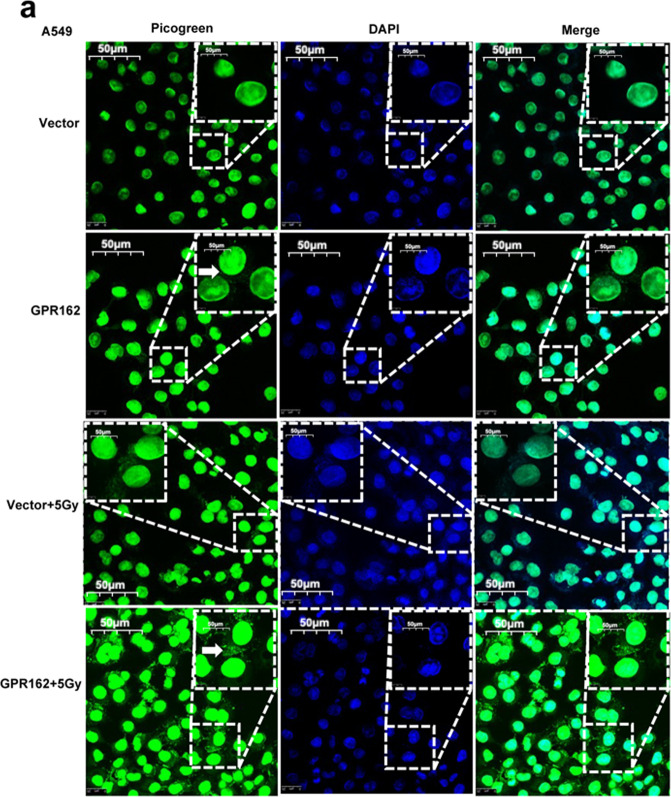
Fig.1a

We apologize for this inadvertent mistake.
