# Supplementary material for: GPR162 activates STING dependent DNA damage pathway as a novel tumor suppressor and radiation sensitizer
Source: Signal Transduct Target Ther. 2023 Feb 1;8:48. doi: 10.1038/s41392-022-01224-3 (PMC9892510; doi:10.1038/s41392-022-01224-3)
Supplement: Supplementary file 1 — GPR162 activates STING dependent DNA damage pathway as a novel tumor suppressor and radiation sensitizer [file 41392_2022_1224_MOESM1_ESM.docx]

Supplementary Materials for

**GPR162 activates STING dependent DNA damage pathway** **as a novel tumor suppressor and radiation sensitizer**

Yao Long ^1,2,3,4^, Jiaxing Guo^1,2,3,4^, Jielin Chen^1,2,3,4^, Jingyue Sun^1,2,3,4^, Haiyan Wang^1,2,3,4^, Xin Peng^1,2,3,4^, Zuli Wang^1,2,3,4^, WeiWei Lai^1,2,3,4^, Na Liu^1,2,3,4,5^, Long Shu^1,2,3,4^, Ling Chen^1,2,3,4^, Ying Shi^1,2,3,4^, Desheng Xiao^1,2,3,4*^, Shuang Liu^1,2,3,4^^*^, Yongguang Tao^1,2,3,4*^

1. Department of Pathology, Key Laboratory of Carcinogenesis and Cancer Invasion(Ministry of Education); Xiangya Hospital, Central South University, Hunan, 410078, China
2. NHC Key Laboratory of Carcinogenesis of Ministry of Health (Central South University), Cancer Research Institute; School of Basic Medicine, Central South University, Changsha, Hunan, 410078, China
3. Department of Pathology, Xiangya Hospital, Central South University, Changsha, Hunan, 410008, China
4. Hunan Key Laboratory of Tumor Models and Individualized Medicine; Department of Thoracic Surgery, Second Xiangya Hospital, Central South University, Changsha, China
5. Hunan International Scientific and Technological Cooperation Base of Brain Tumor Research, Xiangya Hospital, Central South University, Changsha, Hunan, 410008, China

* Corresponding author.

Yongguang Tao. Email: taoyong@csu.edu.cn; Y.T. Key Laboratory of Carcinogenesis and Cancer Invasion, Ministry of Education, Xiangya Hospital, Central South University, Hunan, 410078 China. ORCID ID: 0000-0003-2354-5321. Tel. +(86) 731-84805448; Fax. +(86) 731-84470589.

Shuang Liu. Email: shuangliu2016@csu.edu.cn. Xiangya Hospital, Central South University.

Desheng Xiao. Email: [xdsh96@csu.edu.cn](mailto:xdsh96@csu.edu.cn) Xiangya Hospital, Central South University.

Disclosure of Potential Conflicts of Interest

The authors have declared that no conflict of interest exists.

**This PDF file includes:**

Figures. S1 to S9

Tables. S1 to S5

**Figure. S1.**

**
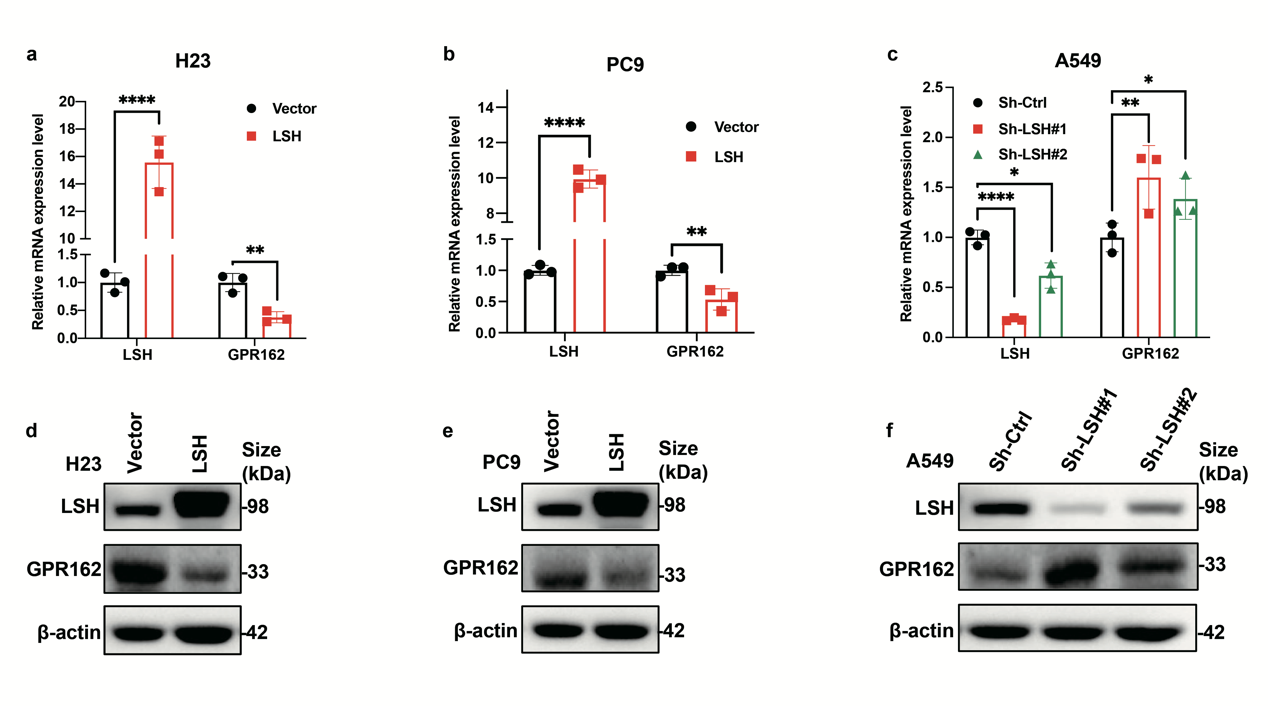
The effect of LSH on the expression level of GPR162 protein and mRNA.**

**a-c** GPR162 mRNA levels in H23 (a), PC9 (b), and A549 (c) cells after LSH overexpression or deletion. **d-f** GPR162 expression was measured by western blot in H23 (d), PC9 (e), and A549 (f) cells after LSH overexpression or depletion. (*p < 0.05, **p < 0.01, ****p < 0.0001)

**Figure. S2.**

**GPR162 interacted with STING at the protein level but did not affect the regulation of downstream transcription level.**

**
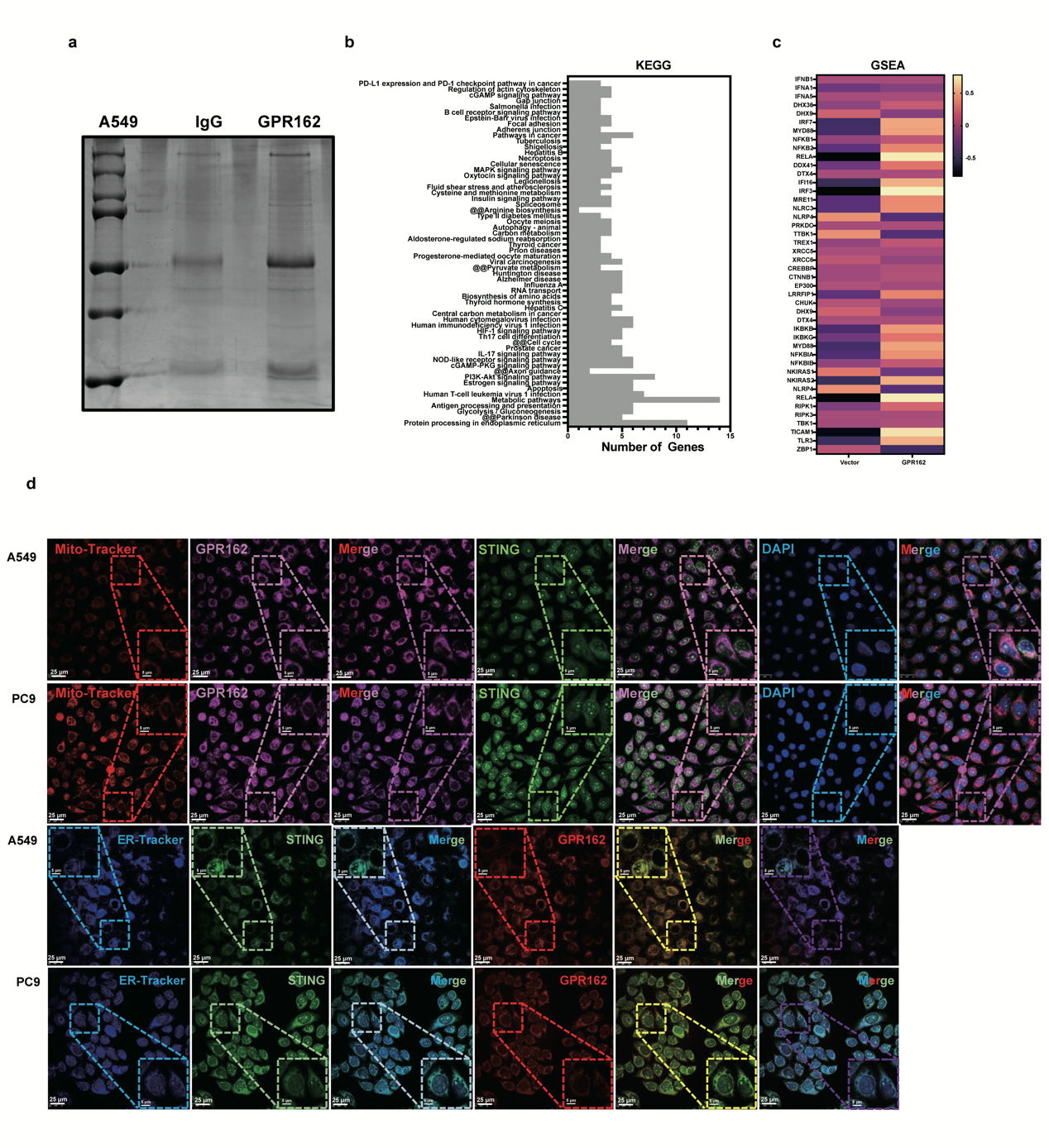
a** The potentially interacting proteins with GPR162 were subjected to a mass spectrometry assay. **b** Proteins interacting with GPR162 were enriched in KEGG. **c** GSEA enriched and assessed the transcription level of STING downstream molecules following GPR162 overexpression. **d** Confocal microscopy images of A549 and PC9 stained with Mito-Tracker, ER-Tracker, anti-GPR162, and anti-STING antibodies and DAPI. Scale bar, 25 μm.

**Figure. S3.**

**
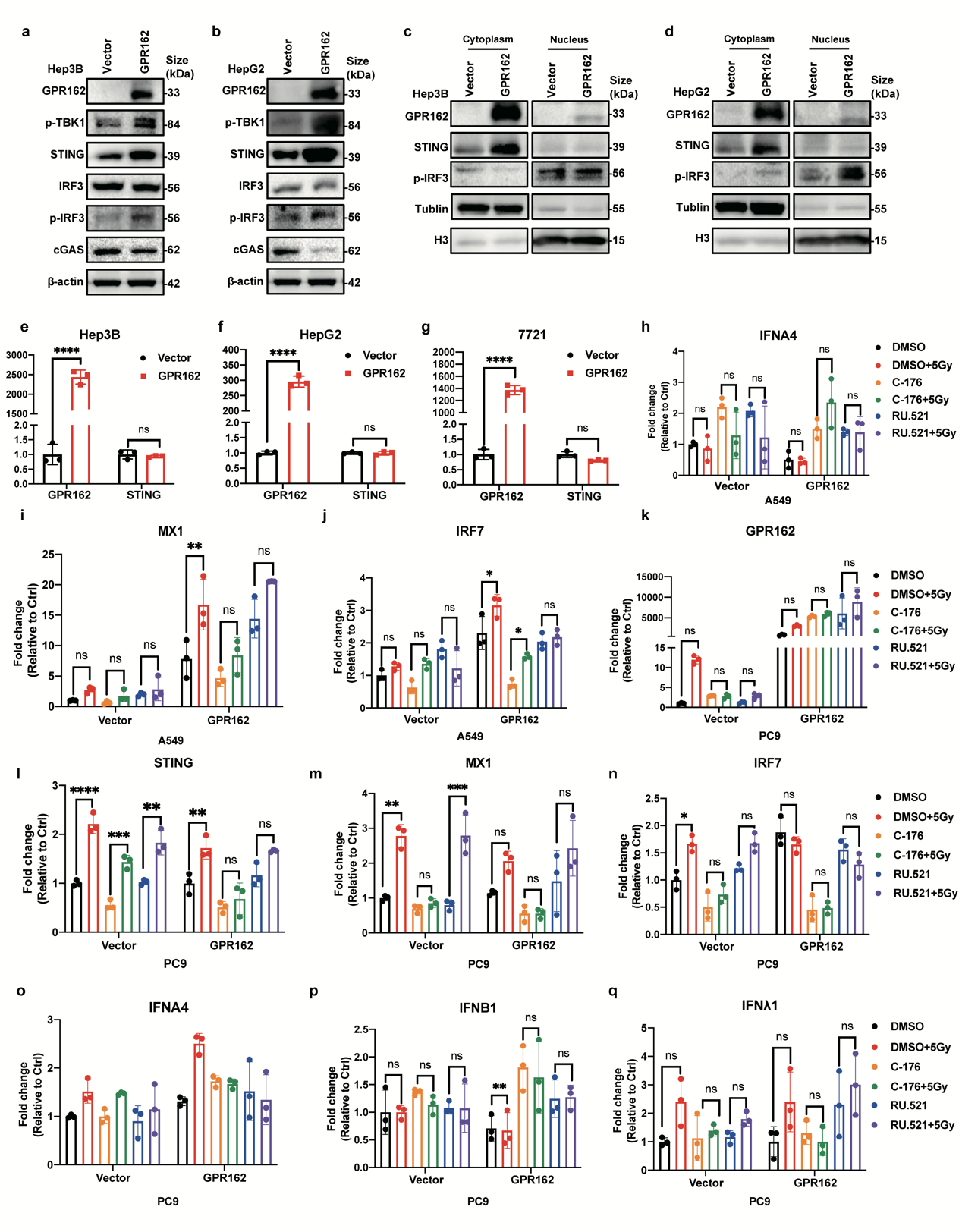
GPR162 activates the STING signaling pathway independent of cGAS.**

**a-b** STING, p-TBK1, p-IRF3, and cGAS expression levels were determined by Western blot analysis in Hep3B (a) and HepG2 (b) cells following GPR162 overexpression. **c-d** STING, p-TBK1, and p-IRF3 expression levels were determined by Western blot analysis in nuclear and cytosolic fractions obtained from Hep3B (c) and HepG2 (d) cells following GPR162 overexpression. **e-g** After overexpressing GPR162, qPCR analyses of STING in Hep3B (e), HepG2 (f), and 7721 (g) cells. **h-q** GPR162, STING, IRF7, MX1, IFNA4, IFNB1, IFN1 qPCR analyses in A549 and PC9 cells treated with IR by cGAS and STING inhibitors following GPR162 overexpression. (*p < 0.05, **p < 0.01, ***p < 0.001, ****p < 0.0001)

**Figure. S4.**

**GPR162 involves in the DNA damage pathway and is more sensitive to DNA damage response.**

**
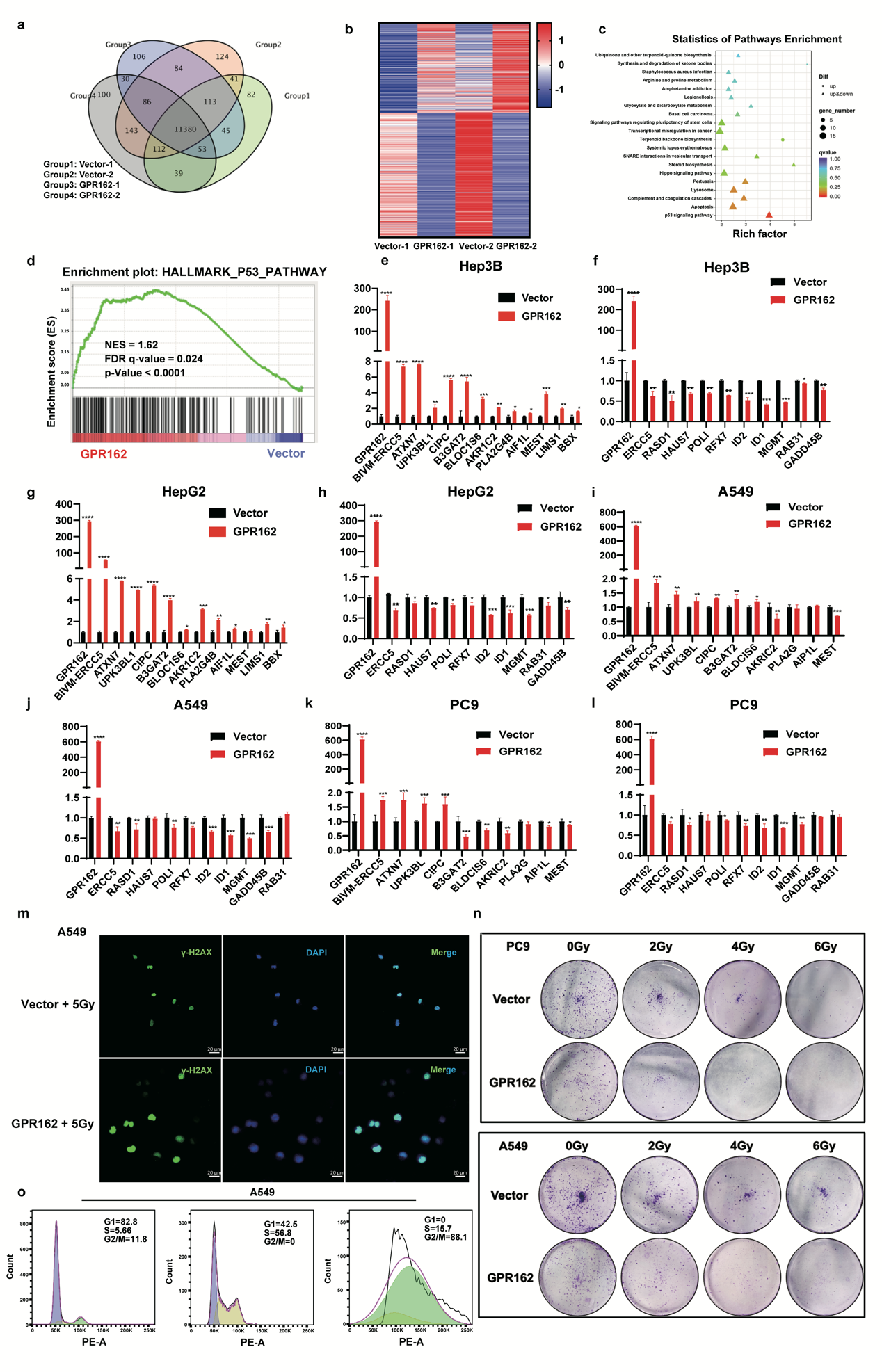
**

**a** Cluster analysis of RNA-Seq findings in GPR162 overexpressed cells was done using a Venn diagram. **b** Heatmap of the differentially expressed genes after the overexpression of GPR162 in A549 cells via RNA-seq. **c** Differentially expressed genes' KEGG enrichment analysis. **d** GSEA of the whole transcriptome. **e-l** The mRNA levels of DNA damage response-related genes were examined by RT-qPCR in Hep3B (e, f), HepG2 (g, h), A549 (i, j), and PC9 (k, l) cells. **m** The fluorescence intensity of γH2AX in A549 cells overexpressing GPR162 following irradiation was observed using confocal microscopy. Scale bar, 20 μm. **n** A colony formation assays using radiation therapy in plates were performed to determine the colony formation ability of A549 and PC9 cells that were stably overexpressing GPR162. **o** The A549 cell cycle was synchronized in the G1 phase, G1/S phase, and G2/M phase by flow cytometry. (*p < 0.05, **p < 0.01, ***p < 0.001, ****p < 0.0001)

**Figure. S5.**

**GPR162 promotes DNA damage response in STING-dependent pathway.**

**
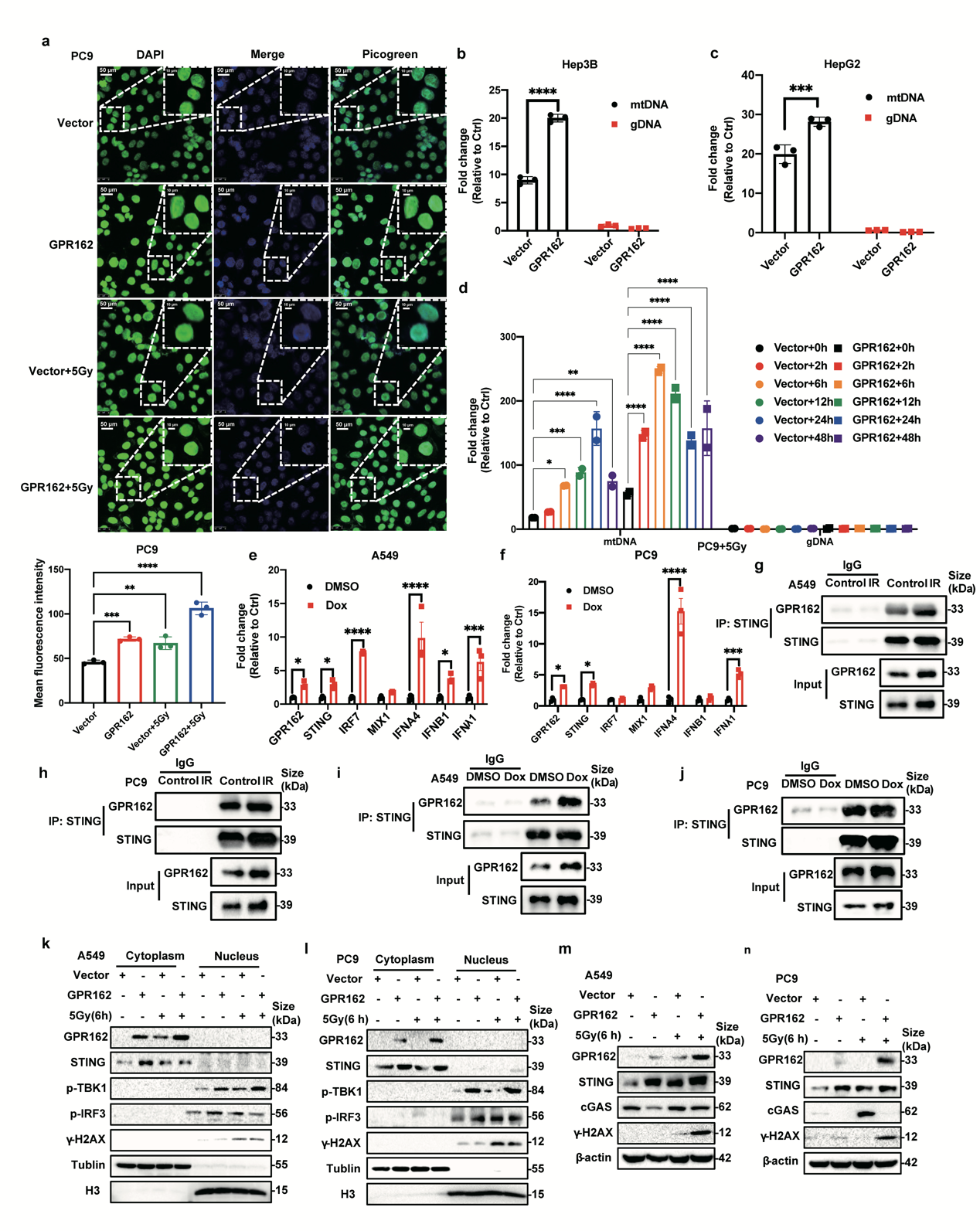
**

**a** Detection of cytosolic DNA in PC9 cells treated with or without IR, as indicated. DNA was detected using the Picogreen fluorescence dye selectively binding dsDNA. Arrows point to cytosolic DNA. Each mean fluorescence intensity of Picogreen was calculated using ImageJ from three different areas. **b-c** qPCR analysis of cytosolic DNA after overexpression of GPR162 in Hep3B (b) and HepG2 (c) cells. **d** qPCR analysis of cytosolic DNA in PC9 cells overexpressing GPR162 after radiotherapy at different time periods. **e-f** qPCR analysis of GPR162 and STING-related genes in A549 (e) and PC9 (f) cells treated with doxorubicin (Dox). **g-j** On A549 (g, i) and PC9 (h, j) cells, IP assays were utilized to examine the interaction between GPR162 and STING after 6 hours of irradiation or 18 hours after doxorubicin (Dox) induction. **k-l** STING, p-TBK1, p-IRF3, and γH2AX protein levels in nuclear and cytosolic fractions induced by IR for 6h were detected by western-blot analysis in A549 (k) and PC9 (l) cells after overexpressing GPR162. **m-n** After overexpression of GPR162, western-blot analysis was utilized to assess the expression levels of GPR162, STING, cGAS, and γH2AX in A549 (m) and PC9 (n) cells induced by IR for 6 hours. (***p < 0.001, ****p < 0.0001)

**Figure. S6.**

**Radiation irradiation can activate STING-related pathways more rapidly in cells overexpressing GPR162.**


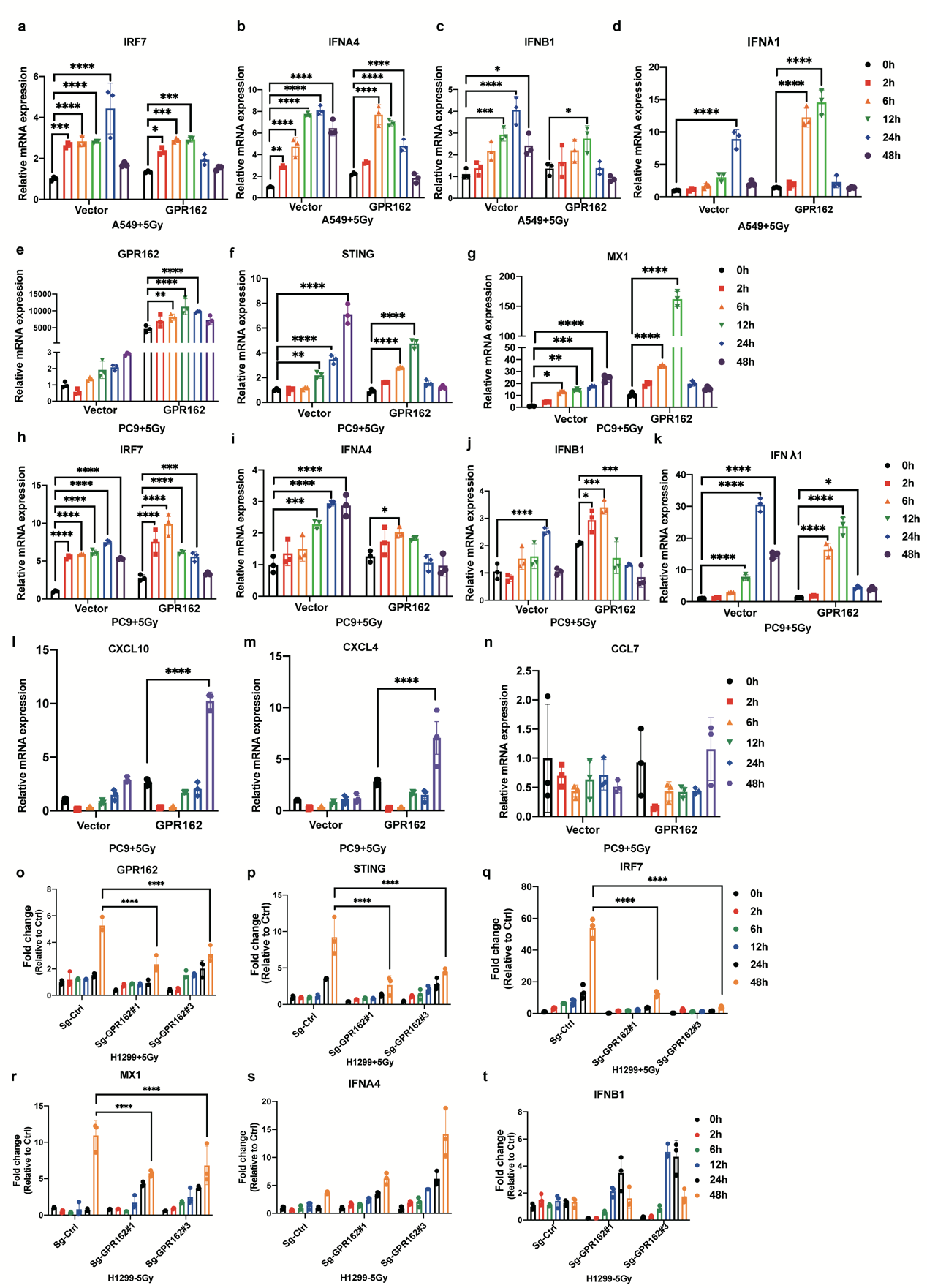


**a-d** qPCR analysis of IRF7 (a), IFNA4 (b), IFNB1 (c), and IFNλ1 (d) mRNA in A549 cells after overexpressing GPR162 induced by IR (5Gy) at different times. **e-n** qPCR analysis of GPR162 (e), STING (f), IRF7 (g), MX1 (h), IFNA4 (i), IFNB1 (j), IFNλ1 (k), CXCL10 (l), CXCL4 (m), and CCL7 (n) mRNA in PC9 cells after overexpressing GPR162 induced by IR(5Gy) with different times. **o-t** qPCR analysis of GPR162 (o), STING (p), IRF7 (q), MX1 (r), IFNA4 (s), and IFNB1 (t) mRNA in H1299 cells after depletion of GPR162 induced by IR(5Gy) with different times. (*p < 0.05, **p < 0.01, ***p < 0.001, ****p < 0.0001)

**Figure. S7.**

**GPR162 is a novel tumor suppressor.**

**
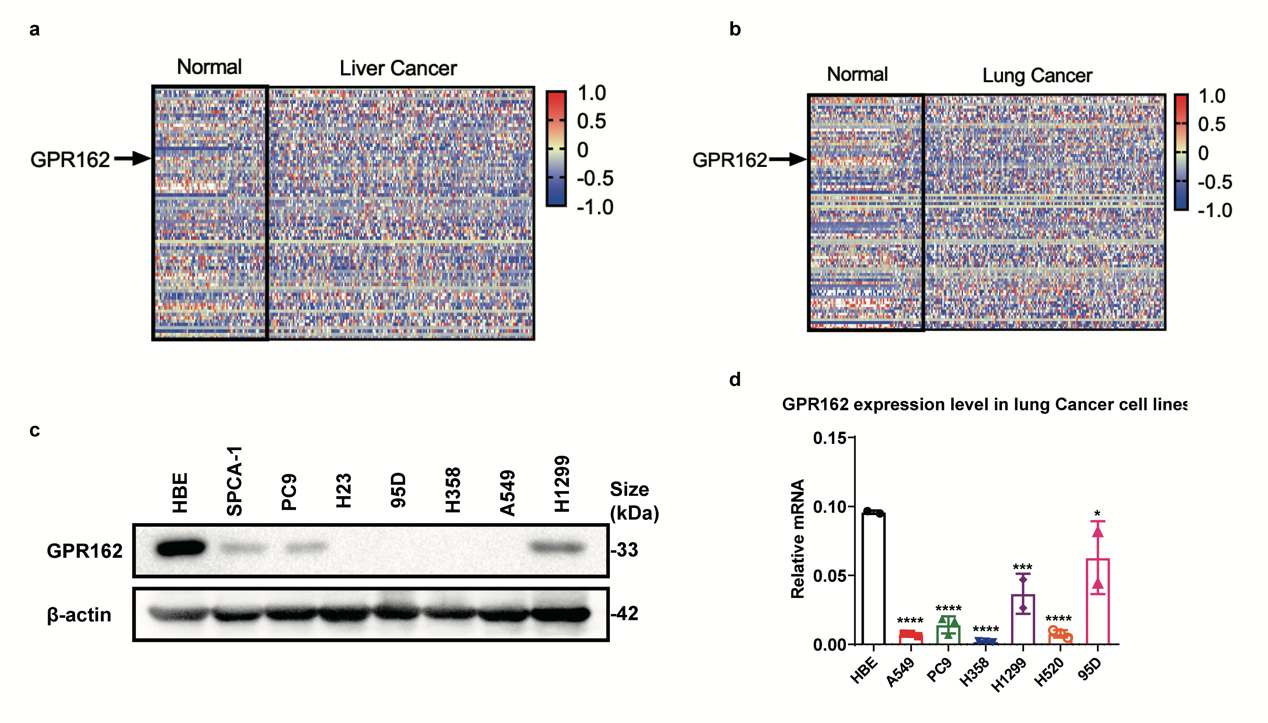
**

**a-b** Heat map of GPCR-related gene expression levels in the lung(a) and liver(b) samples from TCGA. **c** The expression of GPR162 in a panel of lung cancer cell lines was detected using western blot. **d** GPR162 qPCR analysis in lung cancer cell lines. (*p < 0.05, ***p < 0.001, ****p < 0.0001)

**Figure. S8.**

**Images of transwell in cell lines overexpressing GPR162 and images of nude mice xenograft tumors after the injection of cells overexpressing GPR162.**

**
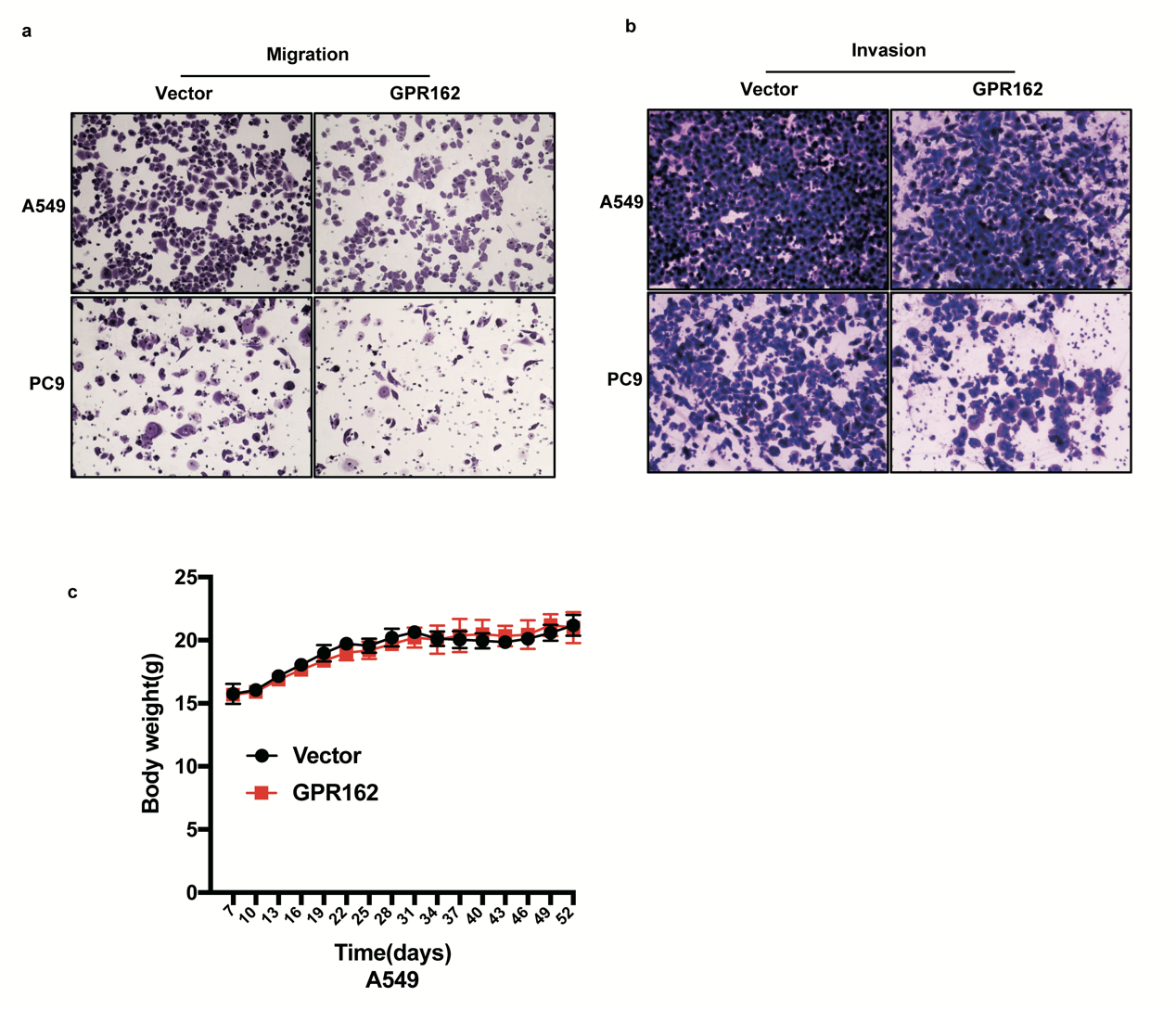
**

**a-b** Representative images of the migration and invasion of GPR162 in A549 cells overexpressing GPR162. **c** The body weight of nude mice after the injection of A549 cells stably expressed control or GPR162 overexpression vectors for 52 days.

**Figure. S9.**

**Images of transwell in cell lines knockout and knockdown GPR162 and images of nude mice xenograft tumors after the injection of cells knockout and knockdown GPR162.**

**
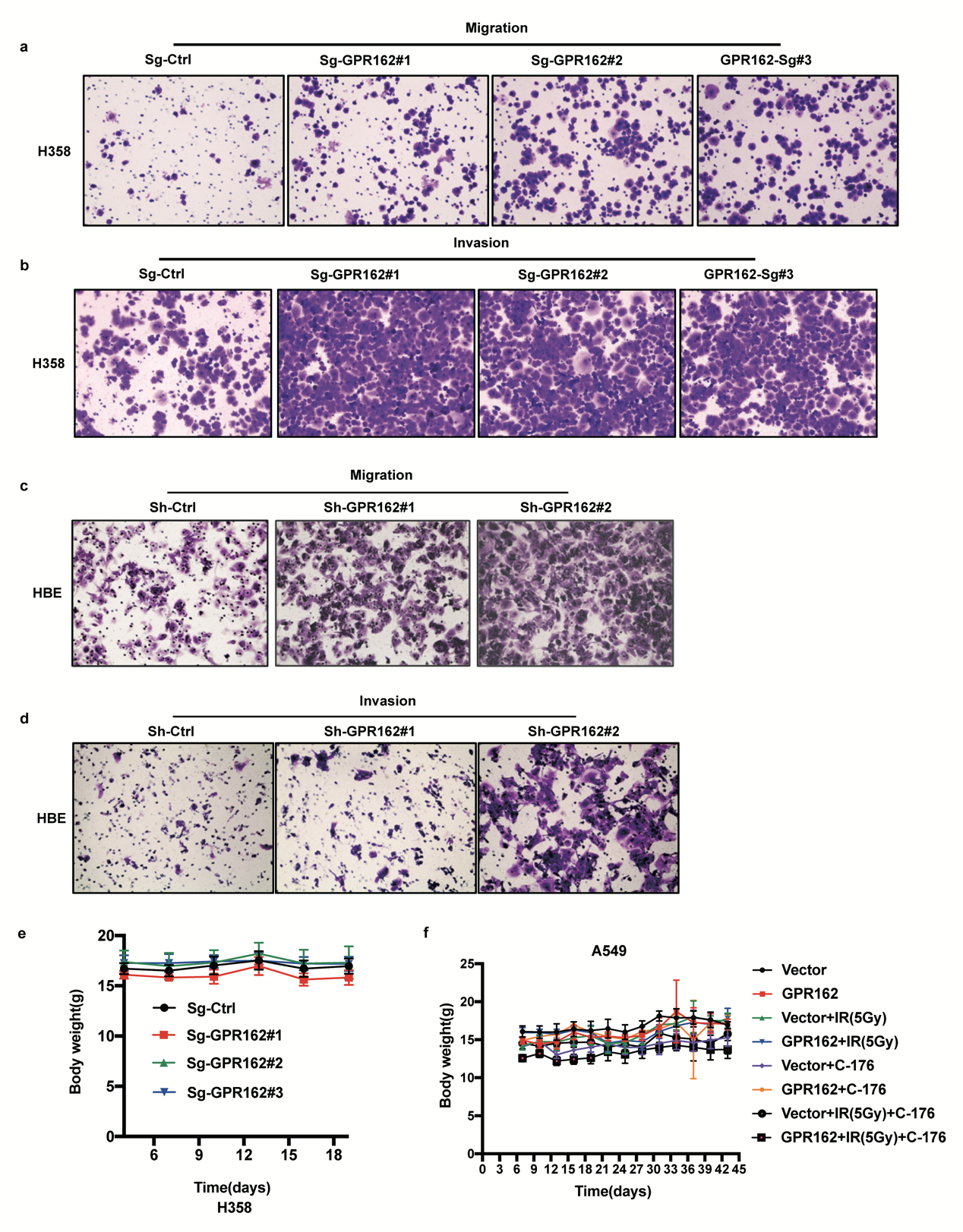
**

**a-b** Representative images of the migration and invasion of GPR162 in H358 cells knockout GPR162. **c-d** Representative images of the migration and invasion of GPR162 in HBE cells knockdown GPR162. **e** The body weight of nude mice after the injection of H358 cells stably expressed control or GPR162 knockout vectors for 52 days. **f** The body weight of nude mice after the injection of A549 cells stably expressed control or GPR162 overexpression for 45 days.

**Table S1.**

**The first 40 proteins that interact with GPR162**

| **Gene names** | **Protein names** |
| --- | --- |
| IGKV A18;IGKV2D-26;IGKV2D-29;IGKV2-40;IGKV2D-30;IGKV2D-28 | Ig kappa chain V-II region RPMI 6410;Ig kappa chain V-II region FR;Ig kappa chain V-II region Cum |
| HSPA9 | Stress-70 protein, mitochondrial |
| HSPA1B;HSPA1A;HSPA8;HSPA2;HSPA6;HSPA7 | Heat shock 70 kDa protein 1B;Heat shock 70 kDa protein 1A;Heat shock cognate 71 kDa protein;Heat shock-related 70 kDa protein 2;Heat shock 70 kDa protein 6;Putative heat shock 70 kDa protein 7 |
| ETFA | Electron transfer flavoprotein subunit alpha, mitochondrial |
| TRIM38 | E3 ubiquitin-protein ligase TRIM38 |
| ATP5A1 | ATP synthase subunit alpha, mitochondrial |
| LDHB | L-lactate dehydrogenase B chain |
| HSPD1 | 60 kDa heat shock protein, mitochondrial |
| PRDX1;PRDX2 | Peroxiredoxin-1;Peroxiredoxin-2 |
| TPI1 | Triosephosphate isomerase |
| PRDX1 | Peroxiredoxin-1 |
| ALDOA | Fructose-bisphosphate aldolase; Fructose-bisphosphate aldolase A |
| CDC42BPG | Serine/threonine-protein kinase MRCK gamma |
| DNAH3 | Dynein heavy chain 3, axonemal |
| ZGPAT | Zinc finger CCCH-type with G patch domain-containing protein |
| SYT9 | Synaptotagmin-9 |
| PHGDH | D-3-phosphoglycerate dehydrogenase |
| **GPR162** | **Probable G-protein coupled receptor 162** |
| EEF2;EFTUD2 | Elongation factor 2;116 kDa U5 small nuclear ribonucleoprotein component |
| FAM83D | Protein FAM83D |
| ASS1 | Argininosuccinate synthase |
| KPNB1 | Importin subunit beta-1 |
| PCMT1 | Protein-L-isoaspartate O-methyltransferase;Protein-L-isoaspartate(D-aspartate) O-methyltransferase |
| LDHA | L-lactate dehydrogenase A chain |
| PC | Pyruvate carboxylase, mitochondrial |
| CDC37 | Hsp90 co-chaperone Cdc37;Hsp90 co-chaperone Cdc37, N-terminally processed |
| HTRA2 | Serine protease HTRA2, mitochondrial |
| HSPD1 | 60 kDa heat shock protein, mitochondrial |
| CCT8 | T-complex protein 1 subunit theta |
| CALR | Calreticulin |
| KHSRP | Far upstream element-binding protein 2 |
| GPI | Glucose-6-phosphate isomerase |
| PAICS | Multifunctional protein ADE2;Phosphoribosylaminoimidazole-succinocarboxamide synthase;Phosphoribosylaminoimidazole carboxylase |
| TRIM28 | Transcription intermediary factor 1-beta |
| POLR3B | DNA-directed RNA polymerase III subunit RPC2 |
| RAN | GTP-binding nuclear protein Ran |
| YWHAE | 14-3-3 protein epsilon |
| MICAL3 | Protein-methionine sulfoxide oxidase MICAL3 |
| NADK | NAD kinase |
| SLC25A11 | Mitochondrial 2-oxoglutarate/malate carrier protein |
| **STING** | **Stimulator of interferon genes protein** |

**Table S2.**

**GPR162_10 Mutations includes 4 duplicate mutations in patients with multiple samples**

| **Sample ID** | **Cancer Type** | **Protein Change** | **Mutation Type** | **Copy #** | **Allele Freq** | **Mut in Sample** |
| --- | --- | --- | --- | --- | --- | --- |
| LUAD-S01478 | Lung Adenocarcinoma | H118N | Missense | Diploid |  | 493 |
| LUAD-NYU408 | Lung Adenocarcinoma | A87D | Missense | Diploid |  | 613 |
| TCGA-17-Z031-01 | Lung Adenocarcinoma | S284R | Missense | Diploid | 0.14 | 2083 |
| TCGA-05-4396-01 | Lung Adenocarcinoma | R218P | Missense | Diploid | 0.02 | 474 |
| TCGA-66-2770-01 | Lung Squamous Cell Carcinoma | R245L | Missense | Diploid | 0.17 | 196 |
| sclc_ucologne_20 | Small Cell Lung Cancer | R245L | Missense | Diploid | 0.32 | 653 |
| TCGA-66-2770-01 | Lung Squamous Cell Carcinoma | R245L | Missense | Diploid | 0.18 | 205 |
| TCGA-66-2770-01 | Lung Squamous Cell Carcinoma | R245L | Missense | Diploid | 0.17 | 196 |
| LUAD-S01478-Tumor | Lung Adenocarcinoma | H118N | Missense | Gain | 0.24 | 405 |
| TCGA-05-4396-01 | Lung Adenocarcinoma | R218P | Missense | Diploid | 0.02 | 445 |

**Table S3.**

**STING_19 Mutations include 11 duplicate mutations in patients with multiple samples**

| **Sample ID** | **Cancer Type** | **Protein Change** | **Mutation Type** | **Copy #** | **Allele Freq** | | **Mut in Sample** |
| --- | --- | --- | --- | --- | --- | --- | --- |
| TCGA-55-7851-01 | Lung Adenocarcinoma | R284M | Missense | Diploid | 0.21 | 126 | |
| TCGA-73-4675-01 | Lung Adenocarcinoma | S4C | Missense | Gain | 0.26 | 63 | |
| TCGA-L9-A8F4-01 | Lung Adenocarcinoma | G90C | Missense | Diploid | 0.38 | 694 | |
| TCGA-18-3409-01 | Lung Squamous Cell Carcinoma | L133F | Missense | ShallowDel | 0.26 | 2458 | |
| TCGA-55-7815-01 | Lung Adenocarcinoma | R248M | Missense | Diploid | 0.22 | 128 | |
| TCGA-73-4675-01 | Lung Adenocarcinoma | S4C | Missense | Gain | 0.26 | 65 | |
| TCGA-18-3409-01 | Lung Squamous Cell Carcinoma | L133F | Missense | Diploid | 0.26 | 2469 | |
| TCGA-46-3768-01 | Lung Squamous Cell Carcinoma | R281L | Missense | ShallowDel | 0.07 | 574 | |
| TCGA-58-8387-01 | Lung Squamous Cell Carcinoma | R169W | Missense | ShallowDel | 0.1 | 145 | |
| TCGA-63-A5MB-01 | Lung Squamous Cell Carcinoma | V113L | Missense | ShallowDel | 0.57 | 307 | |
| TCGA-66-2744-01 | Lung Squamous Cell Carcinoma | Q359K | Missense | Diploid | 0.09 | 329 | |
| TCGA-58-8387-01 | Lung Squamous Cell Carcinoma | R169W | Missense | ShallowDel | 0.11 | 149 | |
| TCGA-55-7815-01 | Lung Adenocarcinoma | R284M | Missense | Diploid | 0.22 | 123 | |
| TCGA-73-4675-01 | Lung Adenocarcinoma | S4C | Missense | Gain | 0.26 | 65 | |
| TCGA-18-3409-01 | Lung Squamous Cell Carcinoma | L133F | Missense | ShallowDel | 0.26 | 2361 | |
| TCGA-63-A5MB-01 | Lung Squamous Cell Carcinoma | V113L | Missense | ShallowDel | 0.6 | 292 | |
| TCGA-L9-A8F4-01 | Lung Adenocarcinoma | G90C | Missense | Diploid | 0.39 | 674 | |
| TCGA-55-7815-01 | Lung Adenocarcinoma | R284M | Missense | Diploid | 0.22 | 122 | |
| TCGA-73-4675-01 | Lung Adenocarcinoma | S4C | Missense | Gain | 0.26 | 63 | |

**Table S4.**

**RT-qPCR primers used for detection of the mRNA level**

| **Name** | **Sequence** | **Product**  **Size(bp)** |
| --- | --- | --- |
| LSH | F:GATTTTGGATCGAATGCTGCCAG  R:ATGGACCCATCAAGCCTGCTGA | 141 |
| GPR162 | F: CTCCTTCATCTGGTCCTGCGAG  R: TGGCTCCGTTAGCATCAAAGCG | 161 |
| β-actin | F: CACCATTGGCAATGAGCGGTTC  R: AGGTCTTTGCGGATGTCCACGT | 135 |
| BIVM-ERCC5 | F: ACACATGCCCAGGGAATTGC  R:GGATTTTCTATTGAGTTCCC | 194 |
| ATXN7 | F: CAACCTCATGGTGGAGAAGCATC  R: ATGTCGGCACAGAGTTTGTCCG | 119 |
| UPK3BL1 | F:CCTGGTGATGAATGACGAAGGAC  R: GGAGGATAGACAGGATGGCGAT | 143 |
| CIPC | F:GAAGAAAGTGGCTCGTCAGCTTG  R: GTCTTCTCTGCTCCAGCCTAAG | 148 |
| B3GAT2 | F: TCTTCGCTGACGACGACAACAC  R: CAACTTTGCCGTTTTCCACCAGC | 138 |
| BLOC1S6 | F: AGTGGAGCAACTGGCAGAAGGA  R:ATCTCTTGTTCCAGTGTGTCTAAC | 123 |
| AKR1C2 | F: CAGTGGATCTCTGTGCCACATG  R: CTGGTTGCAGACAGGCTTGTAC | 149 |
| PLA2G4B | F: GCCTCTTGGATTGCGTCTCCTA  R: CCAGCTTGTTCTTGGTCACCTG | 147 |
| AIF1L | F: CCTTCCAGAAAAGCTCACAGCC  R: CTTCATCTCCAGGTGGGTCTTG | 136 |
| MEST | F: TGTCACCCATCCTCACACGACT  R: GTTCCCGTCATTGTTGCGGATC | 137 |
| LIMS1 | F: TGGTGCGTGAACTGCTTTGCCT  R: CATAGCACTTCTTACAGACTGGC | 103 |
| BBX | F: GTGTCTGCGTTCTTTAGCCTCG  R: TACAGGAGCCTGCGGCATTTCT | 126 |
| ID1 | F:GTTGGAGCTGAACTCGGAATCC  R:ACACAAGATGCGATCGTCCGCA | 145 |
| RFX7 | F: TGCCTTCTGGTCTCAGCAATGG  R: CTGTTTGGGCAGTGAAGTCTCC | 134 |
| ID2 | F: TTGTCAGCCTGCATCACCAGAG  R: AGCCACACAGTGCTTTGCTGTC | 150 |
| ERCC5 | F:GATGCGGAAGATTCGCTCCATG  R: GCTTTCCAGGAACATCTGTCCG | 159 |
| GADD45B | F: GCCAGGATCGCCTCACAGTGG  R:GGATTTGCAGGGCGATGTCATC | 126 |
| POLI | F: CTACTTCACGCTCTGGCAAGCA  R: GTGGTATCTAGTGGAGACTCCC | 145 |
| MGMT | F:CCTGGCTGAATGCCTATTTCCAC  R:GCAGCTTCCATAACACCTGTCTG | 117 |
| HAUS7 | F: CCTTGGCTCAAGGATTCCGTGA  R: CAGAGGTGTCAGCAACTGCCAT | 144 |
| RAB31 | F:GGAGCTGAAAGAACATGGTCCAG  R: GCACTTGTCTCAACCACGATGG | 144 |
| RASD1 | F: CACCGCAAGTTCTACTCCATCC  R: GGTTGTCCAGACTGAACACCAG | 142 |

**Table S5.**

**Plasmid construction primers**

| **Name** | **Sequence** | **Product**  **Size(bp)** |
| --- | --- | --- |
| GPR162-sh#1 | F:CTGTCCCATGATGAGACAAAC | 21 |
|  | R: GTTTGTCTCATCATGGGACAG | 21 |
| GPR162-sh#2 | F:TATGACTGGAACGAGAGTATC | 21 |
|  | R: GATACTCTCGTTCCAGTCATA | 21 |
| GPR162-OE | F:CCGGAATTCATGCTGAGCACTGGGGTGGT | 915 |
|  | R GGACTAGTTCACAGGGTCAGCTGGGGAA |  |
| GPR162-sg#1 | F: CACCGACTCTCGTTCCAGTCATAGT | 25 |
|  | R: AAACACTATGACTGGAACGAGAGTC | 25 |
| GPR162-sg#2 | F: CACCGAAGCGGCGGTCCTCGCTGGA | 25 |
|  | R: AAACTCCAGCGAGGACCGCCGCTTC | 25 |
| cGAS-sh#1 | F:CAACTACGACTAAAGCCATTT | 21 |
|  | R:AAATGGCTTTAGTCGTAGTTG | 21 |
| cGAS-sh#2 | F: ATCTATTCTCTAGCAACTTAA | 21 |
|  | R: TTAAGTTGCTAGAGAATAGAT | 21 |

**Table S6.**

**Antibody**

| **Antibodies** | **Source** | **Identifier** |
| --- | --- | --- |
| Anti-LSH antibody | Santa cruz | Cat. sc-46665 |
| Anti-GPR162 antibody | Proteintech | Cat. 15254-1-AP |
| Anti-STING | Abclonal | Cat. A3575 |
| Anti-p-TBK1-ser172 antibody | Affinity Biosciences | Cat. AF8190 |
| Anti-p-IRF3-S386 antibody | Abclonal | Cat. AP0995 |
| Anti-cGAS antibody | Cell Signaling Technology | Cat. D1D3G |
| Anti-IRF3 antibody | Abclonal | Cat.A19717 |
| Anti-γΗ2ΑΧ antibody | Cell Signaling Technology | Cat. S139 |
| Anti-FLAG antibody | Sigma | Cat. MA1-91878 |
| Anti-β-Actin antibody | Sigma | Cat. A5441 |
| Anti-Tublin antibody | Santa cruz | Cat. 5F131 |
| Anti-H3 antibody | Proteintech | Cat. 17168-1-AP |
| Goat anti-mouse IgG-HRP | Santa Cruz | Cat. sc-2005 |
| Goat anti-rabbit IgG-HRP | Santa Cruz | Cat. sc-2004 |
